# Projected impact of future climate on water-stress patterns across the Australian wheatbelt

**DOI:** 10.1093/jxb/erx368

**Published:** 2017-11-25

**Authors:** James Watson, Bangyou Zheng, Scott Chapman, Karine Chenu

**Affiliations:** 1 The University of Queensland, Queensland Alliance for Agriculture and Food Innovation (QAAFI), Australia; 2 CSIRO Agriculture and Food, Australia

**Keywords:** Climate change, crop adaptation, crop model, drought, environment characterization, environment classification, global warming, water deficit, wheat

## Abstract

Drought frequently limits Australian wheat production, and the expected future increase in temperatures and rainfall variability will further challenge productivity. A modelling approach captured plant×environment×management interactions to simulate water-stress patterns experienced by wheat crops at representative locations across the Australian wheatbelt for 33 climate model projections, considering the ‘business as usual’ emission scenario RCP8.5. The results indicate that projections of future water-stress patterns are region specific. Significant variations in projected impacts were found across climate models, providing local ranges of uncertainty to consider in planning efforts. Most climate models projected an increase in the frequency of severe water-stress conditions in the Western area, the largest producing region, and fewer severe water stresses in other regions. Where found, reductions in water-stress conditions were largely due to shorter crop cycles (a result of warmer temperatures), increased water use efficiency (resulting from increased CO_2_ levels), and, in some cases, increased local rainfall. Overall, simulations indicate that all areas of the Australian wheatbelt will continue to experience severe water-stress conditions (43.9, 42.6, and 40.2% for 2030, 2050, and 2070 compared with 42.8% for 1990). Given projected frequencies of severe water stress and warmer conditions, efforts towards maintaining or improving yields are essential.

## Teaser

The expected increase in temperatures and rainfall variability will challenge crop productivity. Unexpectedly, the frequency of severe water stress is projected to decrease in major wheat-producing regions, while increasing in others.

## Introduction

Demand for staple foods such as wheat continues to increase due to the combination of increasing population and rising living standards (Borlaug and Dowswell, 2005; Godfray *et al.*, 2010). The challenge of meeting this demand is increased by climate change, which has the potential to impact crops through increased temperature and rainfall variability this century (e.g. Howden *et al.*, 2007; Fereres *et al.*, 2011; Qureshi *et al.*, 2013; IPCC, 2014). Given the 5–15 year lead times to produce new cultivars, timely assessments of the impacts of climate change are of crucial importance (Chapman *et al.*, 2012).

Australia produces up to 22 Mt of wheat over 13.5 Mha of land, the majority frequently under water-stress conditions (Chenu *et al.*, 2013). Climate change is projected to impact Australian crops through increased temperature, increased CO_2_, and increased rainfall variability (e.g. Zheng *et al.*, 2012; Potgieter *et al.*, 2013; Reisinger *et al.*, 2014). All these factors can impact water-stress patterns that crops experience. Increased temperatures accelerate crop development and are projected to lead to a shorter crop cycle in the future (Zheng *et al.*, 2012), with possible avoidance of late season water stress; increased CO_2_ fertilizes the crops and will most probably benefit future wheat crops, in particular with greater water use efficiency (Gifford and Morison, 1993; Reyenga *et al.*, 1999; van der Kooi *et al.*, 2016); and increased rainfall variability affects the amount of water available to crops at different times and will have an impact on stress patterns. As both the degree and timing of water stress influence crop growth (e.g. Fischer, 2011), assessing the impact of water deficit on wheat requires careful consideration of the complex interactions between the crop genotype, the local environment, and management practices (G×E×M) (Chenu, 2015). For instance, the timing of sowing, the cultivar chosen (e.g. maturity type), and the timing and intensity of temperature and rainfall all impact on whether or not a crop will be affected by drought.

Process-based crop models have been developed to account for G×E×M interactions (Chenu *et al.*, 2017) and can assist in characterizing both the timing and intensity of stress impacting crops (Chenu, 2015). In addition, crop models may be applied to multisite long-term characterization, for both past and projected future conditions, provided that the model can properly deal with these future conditions. Over the last 15 years, crop models have been used to characterize stress such as drought, heat, and nitrogen stress in many crops (e.g. wheat, barley, sorghum, maize, rice, field pea, and chickpea) in various regions including Australia, Europe, Brazil, China, and India (Chapman *et al.*, 2000; Chapman, 2008; Heinemann et al., 2008, 2011, 2015; Chenu *et al.*, 2009*b*, 2011, 2013, Sadras *et al.*, 2012; Chauhan *et al.*, 2013, 2014, 2017; Kholova *et al.*, 2013; Challinor *et al.*, 2014; Harrison *et al.*, 2014; Chenu, 2015; Lake *et al.*, 2016; Ly *et al.*, 2017). While these studies focused on characterizing local, regional, or national stress for short- or long-term climates, there is a need to better understand projected stress for future climate scenarios, to assist the development of adaptation policies and solutions.

The primary aims of this study were to assess likely changes in water-stress conditions across the wheatbelt, considering both (i) the range of projections from a large ensemble of state-of-the-art climate models, and (ii) consensus that arose from this model ensemble. Projected changes were analysed for ‘viable’ crops only (i.e. crops sown and harvested), with absence of planting opportunities and crop failures analysed separately. In this study, wheat crops were simulated for 60 representative sites across the Australian wheatbelt using the APSIM-Wheat model (Holzworth *et al.*, 2014) for the historical climate (1967–2013, i.e. 1990 baseline) and compared with future scenarios. Heat impacts on grain number and grain weight (e.g. Lobell *et al.*, 2015) were not included in this research as they have not been properly validated yet and do not impact water-stress patterns. Future climate scenarios were generated for 2030, 2050, and 2070 using the projections of 33 climate models from the Coupled Model Intercomparison Project Phase 5 (CMIP5; Taylor *et al.*, 2012), with the ‘business as usual’ Representative Concentration Pathway (RCP) 8.5 scenario. Crop model outputs were used to identify key seasonal patterns of water stress, representing major ‘water-stress environment types’ (Chenu *et al.*, 2011, 2013). The projected trends in water-stress patterns were analysed across all 33 climate models by employing high-performance computing facilities. In addition, ‘optimistic’, ‘central’, and ‘pessimistic’ models were chosen based on their impact on water stress, rather than on standard climatic criteria, to illustrate the range of projected scenarios. Results are presented locally, regionally, and nationally for the different time scales.

## Materials and methods

### Characterizing water-stress environment types across the Australian wheatbelt

**Fig. 1.**
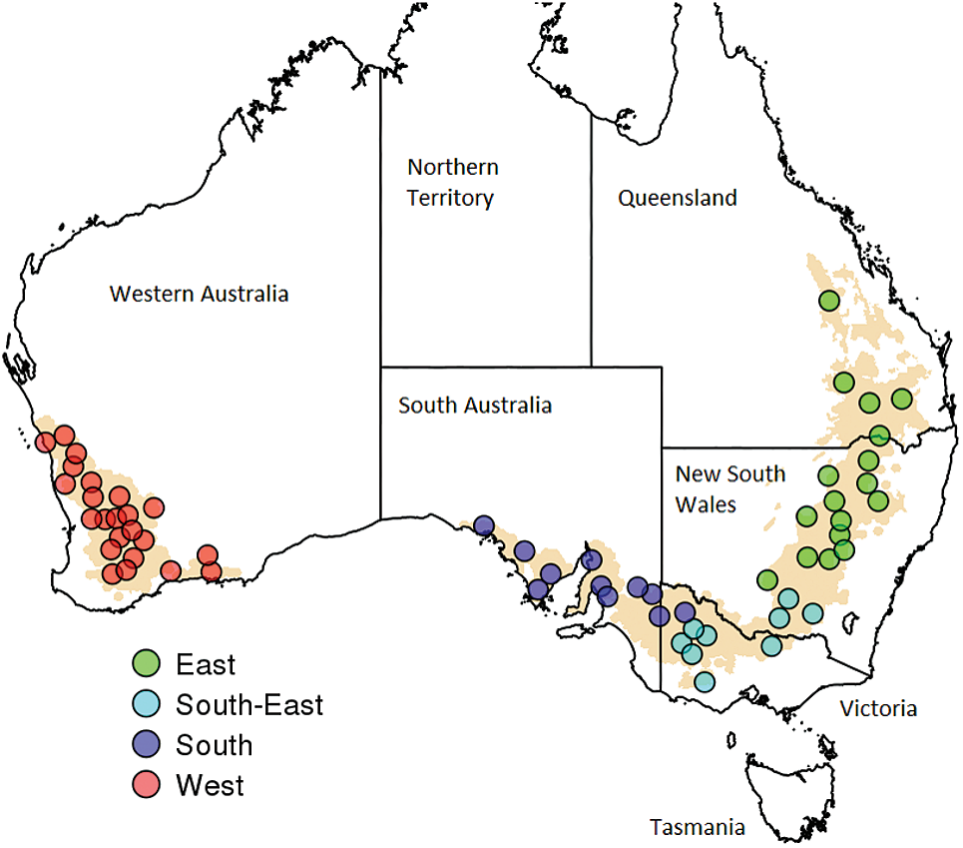
Map of the Australian wheatbelt (pale orange) with the 60 representative locations studied (Supplementary Table S1). The four main wheat cropping areas are indicated by the different colours (green, East; cyan, South-East; dark blue, South; red, West).

Simulations of the broadly adapted, quick–medium maturity wheat variety ‘Hartog’ (*Triticum aestivium* L.) were performed using the Agricultural Production Systems Simulator (APSIM Version 7.6; Holzworth *et al.*, 2014), which has been widely tested for wheat across Australia (e.g. Wang *et al.*, 2009; Chenu *et al.*, 2011; Holzworth *et al.*, 2014; Christopher *et al.*, 2016). In this study, 60 sites were chosen to represent the Australian wheatbelt such that each location represented between 130 000 ha and 230 000 ha of planted wheat (averaged data from 1975 to 2000, 2005, and 2006; source: Australian Bureau of Statistics) (Fig. 1; Supplementary Table S1 at *JXB* online; Chenu *et al.*, 2013).

**Fig. 2.**
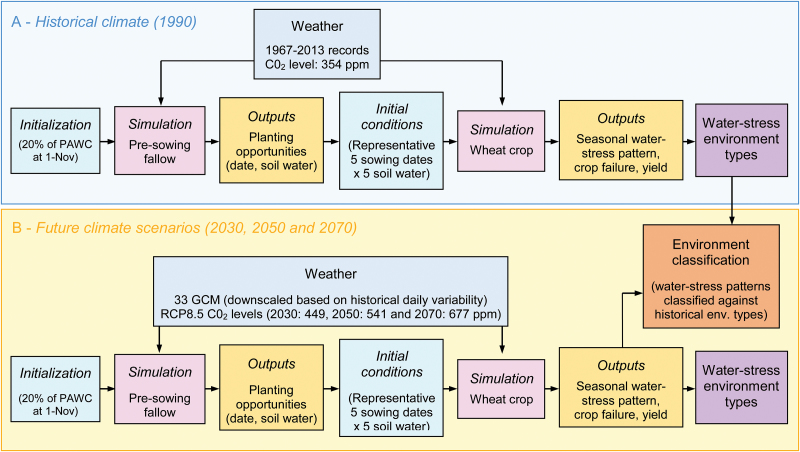
Flow diagram of simulations carried out to characterize the nature and occurrence of water-stress patterns in past (A) and future (B) climate scenarios. For each climatic scenario, a first set of simulations was performed to characterize sowing opportunities and identify representative conditions at sowing (soil water and sowing dates). Using these representative conditions at sowing, a second set of simulations allowed the characterization of seasonal water-stress patterns, crop failure, and yield at each site (Fig. 4: Supplementary Fig. S8). Historical simulations were performed for 1967–2013 weather records, while projected future climates were based on outputs from 33 climate models for 2030, 2050, and 2070 (Table 1, Figs 5, 6; Supplementary Figs S1, S2). Projected monthly outputs from climate models were downscaled to a daily time step by scaling monthly projections by daily variability observed historically. As part of a sensitivity analysis, a daily downscaling with altered rainfall pattern was also tested (not shown on the diagram). The CO_2_ concentrations of Representative Concentration Pathway (RCP) 8.5 scenarios were used (i.e. CO_2_ concentrations of 449, 541, and 677 ppm for 2030, 2050, and 2070). As part of a sensitivity analysis to estimate the CO_2_ impact, the simulations were also performed with baseline scenario (i.e. CO_2_ concentration of 354 ppm for 1990; Fig. 9; not shown on the diagram). Each set of simulations was carried out for 60 sites (Fig. 1; Supplementary Table S1) each represented between 130 000 ha and 230 000 ha of planted wheat. Soil characteristics and management rules for each site were chosen to be representative of the region. Water-stress patterns were output as the daily water-stress index which reflected the ratio of the crop-available soil water to the amount of water that the crop could use for potential transpiration. They were clustered to identify representative water-stress patterns (‘environment types’) in each tested scenario (Fig. 3). Given the similarity among environment types across climatic scenarios, annual water-stress patterns were all classified against historical environment types, thus allowing comparison across scenarios (Figs 7, 8; Supplementary Figs S5–S7).

Each site was defined by a set of management rules, soil characteristics, and historical weather data. Soil characteristics and local management rules (including crop fertilization) were obtained from Chenu *et al.* (2013), which resulted from extensive consultations with local agronomists. The historical weather data for 1967–2013 were obtained from SILO (Jeffrey *et al.*, 2001), and included daily minimum and maximum temperature, solar radiation, and rainfall. As 1990 is commonly used as a reference in future climate studies, the historical time period was selected to be centred on 1990, and extended to recently available data (i.e. 1967–2013). Accordingly, the CO_2_ level of 1990 (i.e. 354 ppm) was used. Two sets of simulations were performed (Fig. 2A). The first set was performed to identify representative initial conditions at sowing (to consider the impact of pre-sowing soil and climate characteristics), while the second set of simulations used those representative initial conditions to identify the main water-stress patterns experienced by wheat crops.

In the first set of simulations, each simulation provided a set of wheat planting conditions (i.e. dates and soil water contents). Simulations were conducted for a fallow between 1 November (the end of the previous crop) and the first sowing opportunities. Simulations assumed that the soil contained 20% of its potential available soil water capacity (PAWC) at the beginning of the fallow (1 November). Note that this initial soil water content at 1 November did not substantially impact on the soil water content at sowing (data not shown). Sowing windows were from 15 April to 15 June in Emerald (Queensland) or from 1 May to 1 July in all other sites. Planting events were defined to occur as in Chenu *et al.* (2013): (i) in the East, if rainfall over the preceding 10 d was at least 10 mm; (ii) in the South and South-East, if 10 d rainfall was at least 5 mm; or (iii) in the West, if rainfall was greater than a threshold defined by a linear decrease over time (from a minimum of 20 mm over 3 d for 1 May, to a minimum of 5 mm over 3 d for 1 July). In addition, a minimum of available soil water was required for the soils of regions with summer-dominant rainfall: 50 mm for Coonamble, Dubbo, Gilgandra, Merriwagga, Urana, Wellington, and Yanco; 80 mm for Condobolin, Dalby, Emerald, Goondiwindi, Gunnedah, Meandarra, Moree, Narrabri, Nyngan, Parkes, Roma, Wagga Wagga, and Walgett; in the rest of the wheatbelt, sowing could occur without any requirement on the soil moisture. A maximum of three potential planting dates were recorded for each year simulated, along with the corresponding available soil moisture. For each site, five soil water conditions and five sowing dates, each representing 20% of the local soil water conditions and sowing opportunities, were consequently identified.

**Fig. 3.**
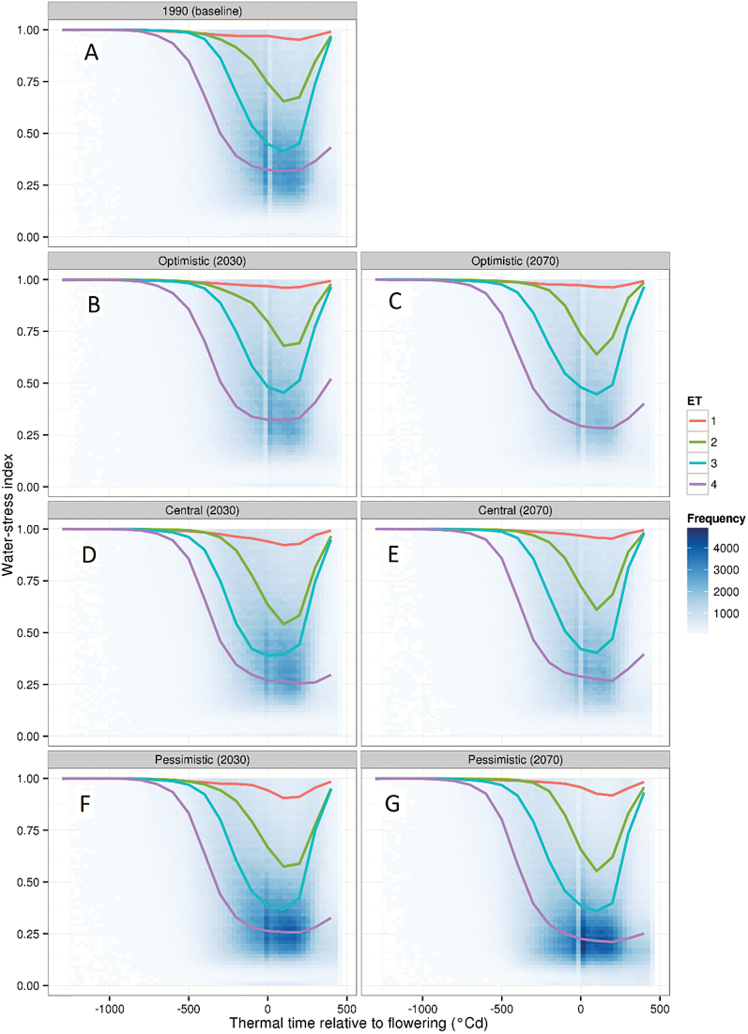
Simulated water-stress index for Australian wheat crops (heat map) and the four main environment types (lines) identified for the baseline historical data (A), optimistic climate model (*CESM1-BGC*; B and C), central climate model (*MRI-CGCM3*; D and E), and pessimistic climate model (*GFDL-ESM2M*; F and G). Results are shown for the time periods 2030 (B, D, and F) and 2070 (C, E, and G). The stress-index corresponds to the ratio of the crop-available soil water to the amount of water that the crop could use for potential transpiration, and ranged from 0 (no water available to the crop) to 1 (no water stress). The water-stress environment types derived from climate scenarios are shown as coloured lines (ET1, orange; ET2, green; ET3, blue; ET4, purple). The frequency of occurrence of water-stress values with respect to thermal time relative to flowering is presented as a heat map, without consideration of the non-stressed value of 1.

The resulting 5 × 5 initial conditions for each site were used in the second set of simulations, to characterize the water-stress patterns experienced by wheat across the wheatbelt. For each initial condition, a crop model simulation was performed to compute a daily water-stress index (Chenu *et al.*, 2013). This index reflected the ratio of the crop-available soil water to the amount of water that the crop could use for potential transpiration, and ranged from 0 (no water available to the crop) to 1 (no water stress). This stress index thus accounts for the plant status (e.g. leaf area and root depth), the weather conditions (e.g. temperature and evaporative demand), and the soil conditions (distribution of the water throughout the soil profile) on the day considered. In other words, this index depends on what happened previously, as the status of the plant and soil are changing daily based on (i) what they were the day before and (ii) the conditions of the considered day. Water-stress patterns for each environment (i.e. each unique site, year, initial soil water, and sowing date combination) were defined by daily water-stress indices centred around flowering and averaged every 100 °Cd, from 100 °Cd after emergence to 450 °Cd after flowering. The resulting water-stress patterns were grouped into four sets using the ‘clara’ partitioning function (Kaufman and Rousseeuw, 1990) to define four major ‘water-stress environment types’ (ET1, ET2, ET3, and ET4; Fig. 3). Each environment (i.e. each unique site, year, initial soil water, and sowing date combination) was assigned to one of the four environment types during the clustering process.

### Projecting future environment types

Monthly outputs of 33 climate models from the CMIP5 (Taylor *et al.*, 2012) were used to generate a set of future climate scenarios (Table 1). These climate models corresponded to the full set of models available in the CMIP5 database when this study began. Downscaling to a daily time step was performed by transforming the local daily historical temperature and rainfall values (1967–2013) by their projected future local monthly means. Three future time periods were studied: centred on 2030 (near-term), 2050 (mid-term), and 2070 (long-term adaptation time frame), with CO_2_ concentrations from the RCP8.5 scenario, which assumes ‘business as usual’ CO_2_ emissions (i.e. CO_2_ concentrations of 449, 541, and 677 ppm for 2030, 2050, and 2070, respectively). Note that precipitation outputs from the climate models were all interpreted as rainfall in this study, as snow is virtually non-existent at the studied sites.

**Table 1.** Seasonal mean temperature and cumulated rainfall projected by each of the 33 member climate model ensemble from the Coupled Model Intercomparison Project Phase 5 (CMIP5). Averages ±SDs were calculated across the 60 studied locations for 1 May to 30 October (i.e. the approximate Australian wheat season). Climate models in bold correspond to the ‘optimistic’ (*CESM1-BGC*), ‘central’ (*MRI-CGCM3*), and ‘pessimistic’ (*GFDL-ESM2M*) models in terms of frequency of severe water stress.

Climate model	Seasonal mean temperature (^o^C)			Seasonal cumulated rainfall (mm)		
	2030	2050	2070	2030	2050	2070
*ACCESS1-0*	14.3 ± 1.7	15.1 ± 1.8	16.1 ± 1.9	230.8 ± 89.9	213.2 ± 88.8	207.6 ± 84.1
*ACCESS1-3*	14.5 ± 1.8	15.4 ± 1.9	16.5 ± 2.0	220.4 ± 88.9	185.4 ± 74.6	151.0 ± 62.9
*bcc-csm1-1*	14.2 ± 1.7	14.8 ± 1.8	15.8 ± 1.8	222.6 ± 85.8	219.2 ± 84.7	193.0 ± 80.1
*bcc-csm1-1-m*	14.8 ± 1.8	15.3 ± 1.8	16.1 ± 1.8	224.3 ± 91.3	217.0 ± 83.7	210.6 ± 83.9
*BNU-ESM*	14.3 ± 1.8	15.2 ± 1.8	16.7 ± 1.8	262.5 ± 102.3	233.4 ± 91.1	175.3 ± 67.9
*CanESM2*	14.5 ± 1.9	15.4 ± 2.0	16.7 ± 2.0	210.3 ± 82.4	203.9 ± 81.2	190.6 ± 74.8
*CCSM4*	14.3 ± 1.8	15.1 ± 1.7	16.1 ± 1.7	230.9 ± 89.9	238.0 ± 93.8	230.4 ± 92.3
***CESM1-BGC***	14.4 ± 1.8	15.0 ± 1.8	16.1 ± 1.8	245.2 ± 96.0	247.7 ± 94.6	224.3 ± 87.7
*CESM1-CAM5*	14.4 ± 1.8	15.1 ± 1.8	16.5 ± 1.9	235.5 ± 92.1	219.0 ± 87.4	208.2 ± 82.0
*CESM1-WACCM*	14.3 ± 1.7	15.1 ± 1.7	15.8 ± 1.7	231.4 ± 90.6	227.9 ± 89.6	229.5 ± 90.8
*CNRM-CM5*	14.0 ± 1.7	14.8 ± 1.8	15.5 ± 1.8	220.1 ± 87.6	215.6 ± 87.5	216.8 ± 85.6
*CSIRO-Mk3-6-0*	14.1 ± 1.8	14.9 ± 1.9	15.9 ± 2.0	198.9 ± 80.6	181.5 ± 74.4	150.7 ± 64.8
*FGOALS-g2*	14.1 ± 1.8	14.6 ± 1.9	15.0 ± 1.8	212.2 ± 84.5	212.5 ± 86.5	254.5 ± 107.2
*FIO-ESM*	14.2 ± 1.7	15.0 ± 1.7	16.2 ± 1.7	232.6 ± 90.9	235.7 ± 92.1	219.7 ± 86.1
*GFDL-CM3*	14.1 ± 1.9	14.9 ± 2.0	16.2 ± 2.2	213.1 ± 83.5	190.6 ± 78.3	153.8 ± 66.3
*GFDL-ESM2G*	14.0 ± 1.8	14.7 ± 1.9	15.4 ± 1.9	215.0 ± 88.5	197.5 ± 82.4	186.9 ± 80.4
***GFDL-ESM2M***	14.3 ± 1.9	14.8 ± 2.0	15.7 ± 2.1	188.1 ± 76.2	165.7 ± 70.0	147.4 ± 62.9
*GISS-E2-H*	14.0 ± 1.8	15.0 ± 1.7	15.9 ± 1.8	229.8 ± 89.0	225.5 ± 89.3	212.8 ± 86.3
*GISS-E2-H-CC*	14.0 ± 1.7	14.8 ± 1.7	15.5 ± 1.7	233.2 ± 93.2	223.0 ± 91.9	227.4 ± 92.5
*GISS-E2-R*	14.1 ± 1.7	14.8 ± 1.8	15.5 ± 1.8	252.6 ± 97.7	239.6 ± 93.8	242.7 ± 95.6
*GISS-E2-R-CC*	14.2 ± 1.8	15.0 ± 1.8	15.9 ± 1.8	228.4 ± 89.1	221.2 ± 87.5	214.7 ± 84.4
*HadGEM2-AO*	14.1 ± 1.8	14.9 ± 1.8	16.0 ± 1.9	230.8 ± 91.3	216.0 ± 87.2	200.2 ± 79.1
*HadGEM2-CC*	14.5 ± 1.7	15.3 ± 1.8	16.1 ± 1.9	219.5 ± 86.9	210.8 ± 83.3	199.3 ± 80.5
*HadGEM2-ES*	14.5 ± 1.7	15.4 ± 1.8	16.4 ± 1.9	231.9 ± 91.3	204.6 ± 80.0	177.7 ± 71.3
*inmcm4*	13.9 ± 1.8	14.5 ± 1.8	15.2 ± 1.9	246.7 ± 97.3	240.0 ± 94.9	220.0 ± 86.2
*IPSL-CM5A-LR*	14.5 ± 1.8	15.4 ± 1.9	16.6 ± 1.9	208.7 ± 86.4	199.9 ± 83.1	180.9 ± 74.6
*IPSL-CM5A-MR*	14.5 ± 1.8	15.4 ± 1.9	16.9 ± 1.9	198.5 ± 79.2	192.1 ± 76.9	159.4 ± 66.1
*IPSL-CM5B-LR*	14.1 ± 1.8	14.8 ± 1.9	15.8 ± 1.9	224.0 ± 87.7	210.2 ± 81.9	201.2 ± 79.2
*MIROC5*	14.1 ± 1.8	14.7 ± 1.8	15.4 ± 1.9	234.0 ± 90.5	221.4 ± 86.2	230.9 ± 90.7
*MIROC-ESM*	14.1 ± 1.6	14.8 ± 1.6	15.8 ± 1.7	242.2 ± 96.4	251.1 ± 103.2	229.3 ± 100.3
*MIROC-ESM-CHEM*	14.1 ± 1.6	14.8 ± 1.6	15.8 ± 1.6	249.6 ± 100.3	251.3 ± 102.5	229.2 ± 97.5
***MRI-CGCM3***	13.9 ± 1.7	14.6 ± 1.6	15.3 ± 1.7	227.5 ± 88.9	224.3 ± 93.4	220.1 ± 93.1
*NorESM1-M*	14.0 ± 1.7	14.8 ± 1.7	15.5 ± 1.7	230.1 ± 91.5	221.3 ± 91.3	233.9 ± 103.2

For each of the 198 future climate scenarios, representative future initial soil water values and sowing dates were identified at each site using the same procedure as for historical data (described above, Fig. 2B). They were used to initialize simulations for each climate scenario×site×year×initial soil water×sowing date combination in order to determine the water-stress patterns experienced by crops. The water-stress index was computed daily, and patterns over time were clustered for each climate scenario using the same method as for historical data (described above; Figs 2B, 3). To allow comparison of stress frequencies across scenarios, and given that the main water-stress patterns did not change substantially across scenarios (Fig. 3), individual water-stress patterns from each environment (i.e. climate×site×year×initial soil water×sowing date combination) were assigned to one of the four historical water-stress environment types (ETs; Fig. 3A). Hence, for each simulation, seasonal patterns were classified based on which historical ET they were most similar to, based on the minimum sum of squared differences for the considered water-stress pattern compared with the water-stress pattern of the historical ET (Fig. 2B).

Projected site-level impacts were analysed for sowing opportunities (i.e. presence/absence of opportunity and potential shifts in sowing date), crop failure, and water-stress patterns. Crops were considered as ‘unviable’ (i.e. crop failure) when yielding <200 kg ha^−1^ as growers would lose money harvesting them. The analysis of water-stress patterns was performed only for viable crops (i.e. where crops were sown and yielded >200 kg ha^−1^).

To reflect the range and potential consensus of projections across climate models, projected site-level impacts are presented both for (i) three climate models that represent optimistic (*CESM1-BGC*), central (*MRI-CGCM3*), and pessimistic (*GFDL-ESM2M*) scenarios with respect to the severe water-stress conditions of ET3 and ET4; and for (ii) the average and SD of impacts across all 33 climate models. Climate models were ranked in terms of their impact on water-stress patterns to identify the optimistic, central, and pessimistic climate models.

The simulations for the 2030, 2050, and 2070 time frames described above were repeated with the baseline CO_2_ level (354 ppm, i.e. CO_2_ level from 1990) to assess the impact of CO_2_ on projected water-stress patterns. In addition, three different methods of introducing extra dry days were tested in order to explore possible bias introduced by retaining historical local rainfall patterns in future climate scenarios. Rainfall patterns were altered by adding extra dry days to each year, with the number of extra dry days interpolated from the CMIP5- and RCP8.5-based projections of Polade *et al.* (2014). In these ‘dry day’ simulations, the rainfall of days selected to become dry was either (i) shifted to the following day, hence maintaining the monthly rainfall totals; (ii) proportionally redistributed to remaining rainy days within the considered month, again without change to the monthly rainfall amount; or (iii) removed altogether.

Overall, 33×3×5 future scenarios of daily local temperature and rainfall were used for each of 60 sites. Over 36.6 million crop simulations were conducted (1 historical scenario+99 future scenarios)×[(60 sites×47 years)+(60 sites×47 years×5 initial soil water values×5 planting dates)]×(2 CO_2_ configurations+3 ‘dry day’ configurations). These simulations were completed using the High Performance Computing facility of the University of Queensland, as it would have taken ~424 d of computing time to run these simulations in serial on similar processors (2.93 GHz Intel Xeon X5570). The analysis was performed using Python (Python Software Foundation) and R (R Core Team, 2015).

## Results

### A projected increase in seasonal temperature across the wheatbelt

**Fig. 4.**
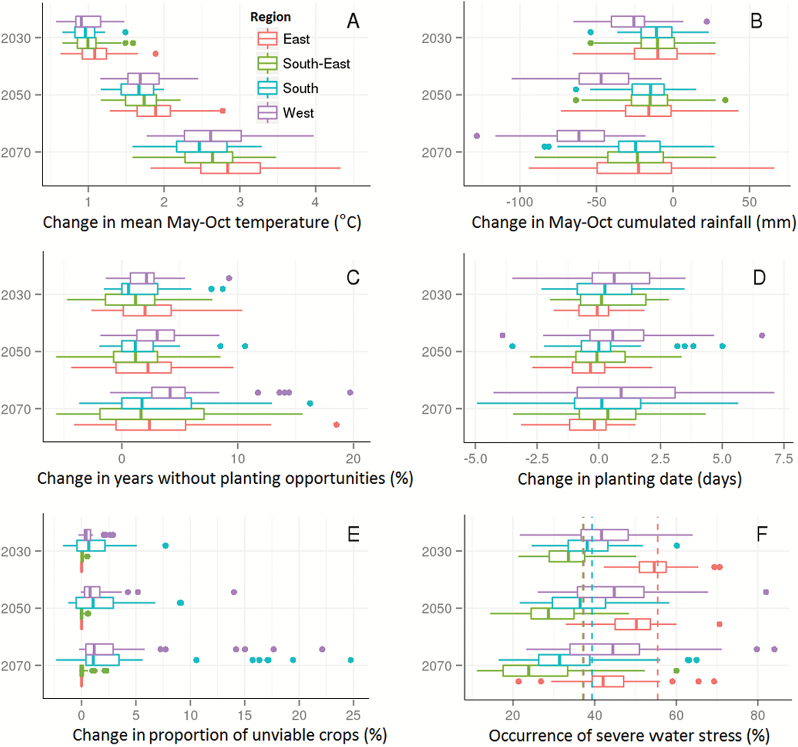
Projected changes in seasonal mean temperature (A), seasonal cumulated rainfall (B), frequency of no planting opportunity (C), planting dates (D), frequency of crop failure (E), and frequency of severe water-stress environment types (F) averaged across sites, seasons, and initial conditions for each of the 33 CMIP5 climate models for 2030, 2050, and 2070. Changes in temperature, rainfall, planting opportunities, planting date, and crop failure relate to differences between the future climate scenarios and the respective baseline (1990) values. The occurrence of severe water stress (F; percentage of occurrence of ET3 and ET4) is given for the 1990 baseline (dashed vertical lines) and future climate scenarios (boxplots). Note that the 1990 dashed lines for the South-East and West overlap. For the boxplots, the line in the middle of the boxes represents the median value for the data, the upper edge of the boxes represents the 75^th^ percentile, and the lower edge represents the 25^th^ percentile. The whiskers correspond to 1.5 times the interquartile range (IQR; the difference between the 75^th^ and 25^th^ percentiles) or to the most extreme observed value, whichever is smallest. Dots outside the whiskers represent individual values outside this range.

**Fig. 5.**
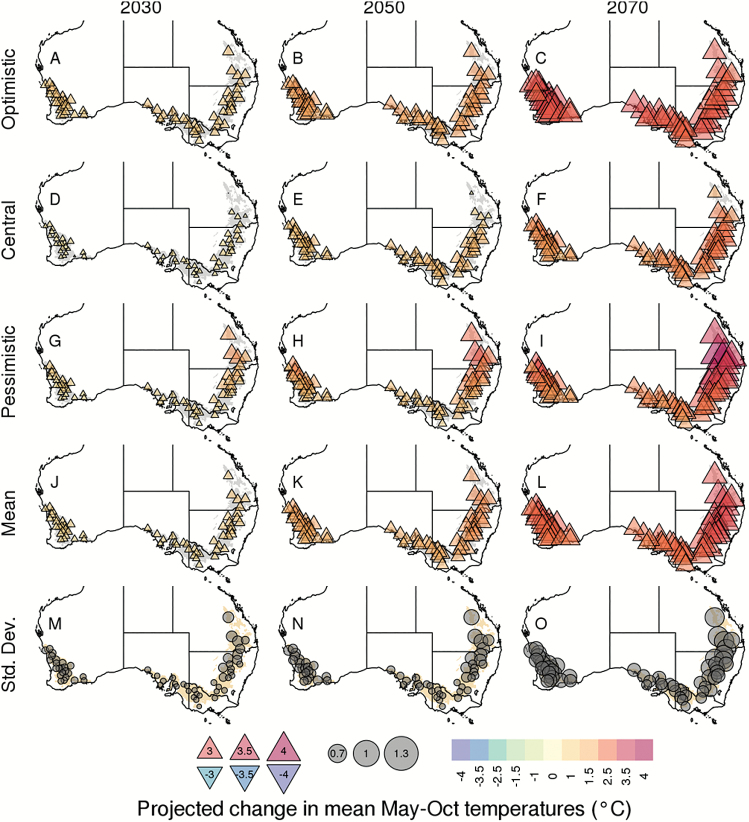
Maps of site-level changes in seasonal mean temperatures projected by the optimistic (*CESM1-BGC*), central (*MRI-CGCM3*), and pessimistic (*GFDL-ESM2M*) climate models, as well as the average and SD across the 33 climate models (rows) for the 2030, 2050, and 2070 time frames (columns). Changes are calculated according to the respective 1990 (baseline) values. Changes in seasonal variations were calculated for 1 May to 31 October, and are presented with symbol size and colour (upright triangles for increase in temperature, inverted triangles for a decrease in temperature, where the size and colour of the triangles indicate magnitude; grey circles for SD). Note the lack of a clear temperature trend across the optimistic, central, and pessimistic climate models, which were selected to represent the range of impacts on severe water stress within the climate model ensemble. See Supplementary Fig. S1 for the absolute projected seasonal mean temperatures.

For the wheat season, climate models exhibited an increase in seasonal temperatures (May to October) from 2030 to 2070 in Australia (Figs 4A, 5). Across the Australian wheatbelt, the projected climate ensemble mean increased by 1.0, 1.8, and 2.7 °C for 2030, 2050, and 2070, respectively. Uncertainty in projections also increased over time, with site SDs increasing with respect to the time frame considered (Fig. 5M–O). Overall, the mean seasonal temperature is projected to be between 13.9 °C (*inmcm4* model) and 14.8 °C (*bcc-csm1-1-m*) in 2030, between 14.5 °C (*inmcm4*) and 15.4 °C (*IPSL-CM5A-LR*) in 2050, and between 15.0 °C (*FGOALS-g2*) and 16.9 °C (*IPSL-CM5A-MR*) in 2070 (Table 1).

Results from the climate models were highly variable across locations (Figs 4A, 5; Supplementary Fig. S1). For instance, 76% of the models projected greater increases in temperature in the East than in the rest of the wheatbelt. On average, seasonal temperatures were projected to increase by 1.2, 2.0, and 3.1 °C in the East compared with 1.0, 1.7, and 2.6 °C for the rest of the wheatbelt by 2030, 2050, and 2070 respectively.

### A projected decrease in seasonal rainfall, especially in the West

**Fig. 6.**
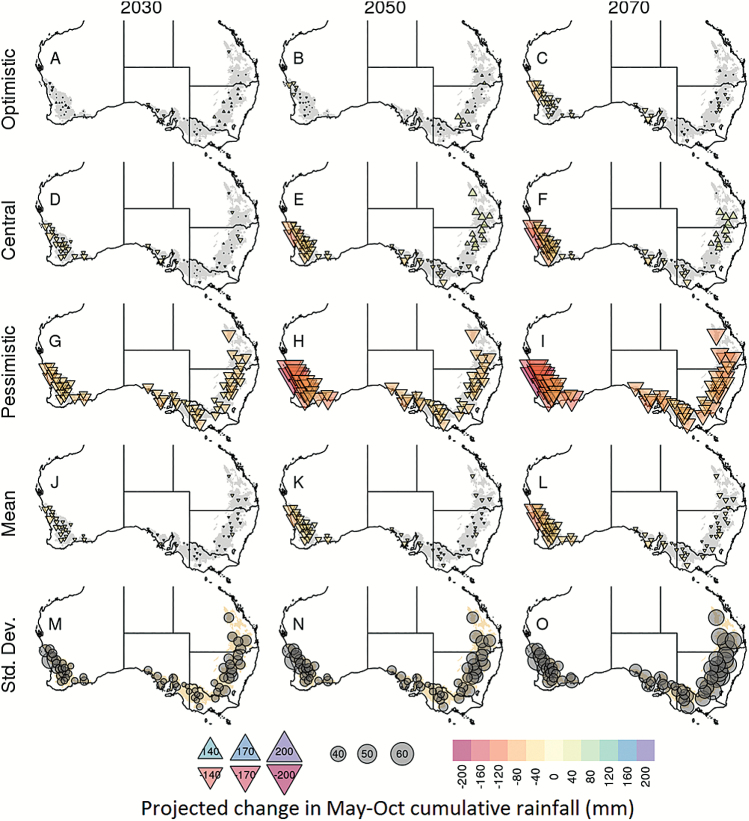
Maps of site-level changes in seasonal cumulated rainfall projected by the optimistic (*CESM1-BGC*), central (*MRI-CGCM3*), and pessimistic (*GFDL-ESM2M*) climate models, as well as the average and SD across the 33 climate models (rows) for the 2030, 2050, and 2070 time frames (columns). Changes are calculated according to the respective 1990 (baseline) values. Changes in seasonal rainfall were calculated for 1 May to 31 October, and are presented with symbol size and colour (upright triangles for increases in rainfall, inverted triangles for decreases in rainfall, with colour and symbol size indicating magnitude; grey circles for SD). See Supplementary Fig. S2 for absolute projected seasonal rainfall for 2030, 2050, and 2070.

In contrast to mean temperature, climate models varied in the direction (positive/negative) of projections for seasonal rainfall compared with the baseline (Figs 4B, 6; Supplementary Fig. S2). For instance, some models predict increased seasonal rainfall in the East (e.g. ‘central’ model, Fig. 6) while others predict a decrease in rainfall in this region (e.g. ‘pessimistic’ model, Fig. 6; Supplementary Fig. S2). On average across models, mean seasonal cumulative rainfall was projected to decrease in the wheatbelt (Fig. 6J–L) by –17.7 mm (i.e. –7.25%) as early as 2030 (and by –11.28% and –16.56% for 2050 and 2070, respectively). Most climate models projected greater decreases for the West than for other regions (Figs 4B, 6), with 26.7, 45.5, and 64.3 mm less rainfall on average in the West by 2030, 2050, and 2070 respectively. The results from altering the daily rainfall patterns (i.e. the three ‘dry day’ configurations detailed in the Materials and methods) did not differ significantly from those derived from the historical rainfall patterns. The number of extra dry days added to change the rainfall pattern (i.e. interpolated at an extra 0.2 d year^–1^; Polade *et al.*, 2014) did not significantly impact the water-stress environment types in the time frames studied. The rest of this paper analyses only the projected rainfall based on historical daily patterns.

### Fewer sowing opportunities, especially in the West

An initial set of APSIM crop simulations were performed for each site and climate scenario to identify potential changes in sowing opportunities (Fig. 4C, D; Supplementary Figs S3, S4), assuming no change in local management practices (i.e. sowing decision based on rainfall patterns, see the Materials and methods, Fig. 2). On average, the number of years with sowing opportunities was projected to decrease slightly in the coming decades (–1.8, –2.1, and –3.7% for 2030, 2050, and 2070, respectively), with greater impact in the West (–2.0, –3.1, and –5.3% for 2030, 2050, and 2070, respectively) than in the rest of the wheatbelt (Fig. 4C). The average sowing date was projected not to change substantially, but tended to be slightly delayed in the West (shift by 0.7, 0.7, and 1.1 d for 2030, 2050, and 2070, respectively). Note that sowing opportunities were partly defined based on the occurrence of rainfall breaks, which may not be well predicted as the projected variability in rainy days was maintained within each month (i.e. projected monthly means were transformed by daily historical observations; Fig. 2).

### No major change in crop viability

Crop viability was defined here as planted crops that yielded >200 kg ha^−1^. With this threshold, only small increases in unviable crops were found across the wheatbelt (0.9 ± 1.4%, 1.6 ± 2.4%, and 3.7 ± 5.8% by 2030, 2050, and 2070, respectively; Fig. 4E). However, in reality, crops with greater yield may be considered as crop failure by growers. When considering failure for a 500 kg ha^−1^ threshold, the projected climatic impact on crop viability was significantly higher, ranging from –2.5% to +61.1% in the West by 2070, depending on the climate model considered (national average of 1.2, 2.2, and 4.8% by 2030, 2050, and 2070, respectively; data not shown). Note that in both cases, virtually no crop failure occurred in the East and South-East, where growers only sow a crop if sufficient soil water is present at the beginning of the season, while in the South and West, crops rely almost entirely on seasonal rainfall, so planting opportunities can occur during more adverse conditions.

### More severe water stress in the West, less severe water stress in the East

Across the wheatbelt, severe water stress (i.e. lower water-stress index in Fig. 3) occurred around flowering and during the grain-filling period, with a frequency that increased from 1990 to 2070 (darker blue in Fig. 3). Occurrences of severe water stress were also projected to be more frequent in the pessimistic, than in the optimistic climate model, as expected.

The timing and intensity of the water-stress index centred around flowering were clustered to identify four main environment types for each climate scenario (coloured lines in Fig. 3). Historical environment types (Fig. 3A) were similar to those previously identified for 1889–2011 (Chenu *et al.*, 2013). In all future climate scenarios, the four main drought patterns were relatively similar to the four historical patterns in terms of the timing and frequency of water stress (coloured lines in Fig. 3): environment type 1 (ET1) represented environments with no or low water stress, environment type 2 (ET2) corresponded to mild post-flowering stresses, environment type 3 (ET3) encompassed environments with stress developing during the vegetative period and relieved during the grain-filling period, and environment type 4 (ET4) represented the most severe stresses, beginning early with no relief. Yield was most affected by the severe ET3 and ET4 stresses.

**Fig. 7.**
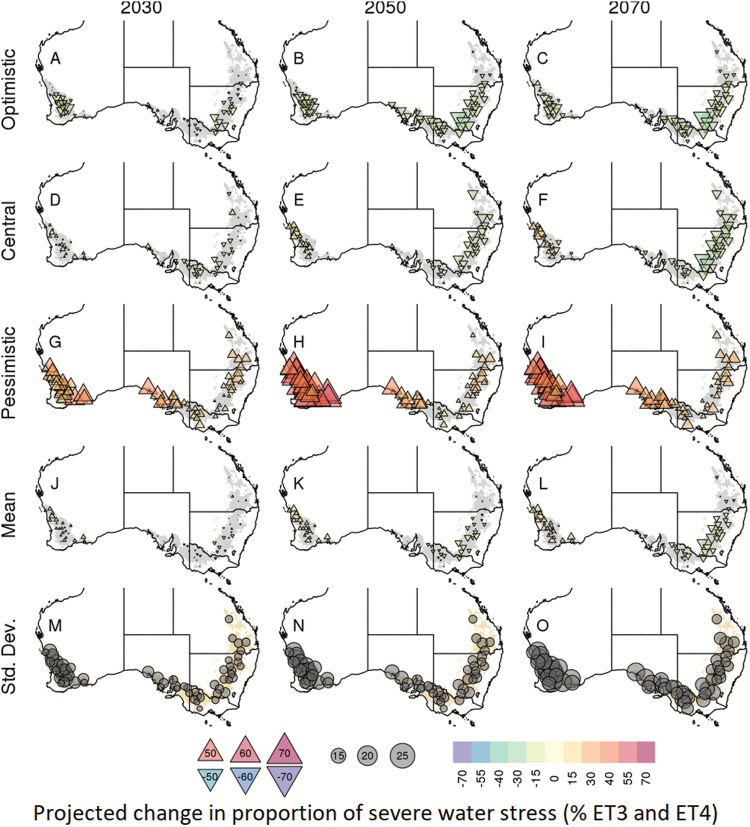
Maps of site-level changes in the occurrence of severe water-stress environment types (ET3 and ET4) projected from the crop simulations based on projected climatic data (Figs 5, 6). The optimistic (*CESM1-BGC*), central (*MRI-CGCM3*), and pessimistic (*GFDL-ESM2M*) climate models, as well as the average and SD across the 33 member climate ensemble, are grouped into rows, and the 2030, 2050, and 2070 time frames are grouped into columns. Changes were calculated according to the respective 1990 (baseline) values. Changes in water-stress frequency are presented by symbols and colour (upright triangles for increases in frequency, inverted triangles for decreases in frequency, with colour and symbol size indicating magnitude; grey circles for SD). See Fig. 8 for the absolute projected occurrences of severe water stress for 2030, 2050, and 2070.

Significant regional differences in the occurrence of severe water stress (environment types ET3 and ET4) were projected by the 33 climate models, with clear differences among these models highlighting a large range of uncertainty in terms of both water stress (Figs 4F, 7) and yield (Supplementary Fig. S8). For instance, increases in water-stress conditions and reduction in yield were identified in the West as early as 2030 for the central (*MRI-CGCM3*) and pessimistic (*GFDL-ESM2M*) climate models, while the optimistic climate model (*CESM1-BGC*) projected a decrease in severe water-stress and an increase in yield in this region for 2030 (Fig. 7A, D, G; Supplementary Fig. S8). In contrast, the optimistic and central climate models projected a decrease in severe water stress conditions in the East, South-East, and South, while the pessimistic model projected an increase in severe water stress in these regions. Across the 33 climate models, severe water-stress projections ranged from decreases of –15.67% (*BNU-ESM*) to increases of 26.72% in 2030 (*GFDL-ESM2M*) and from –14.10% (*GISS-E2-R*) to 46.74% in 2070 (*GFDL-CM3*) in the West (Fig. 4F). Overall, projections in severe water-stress occurrence and yield varied greatly within the climate ensemble studied, as illustrated in Fig. 4F; Supplementary Figs S8, S9.

**Fig. 8.**
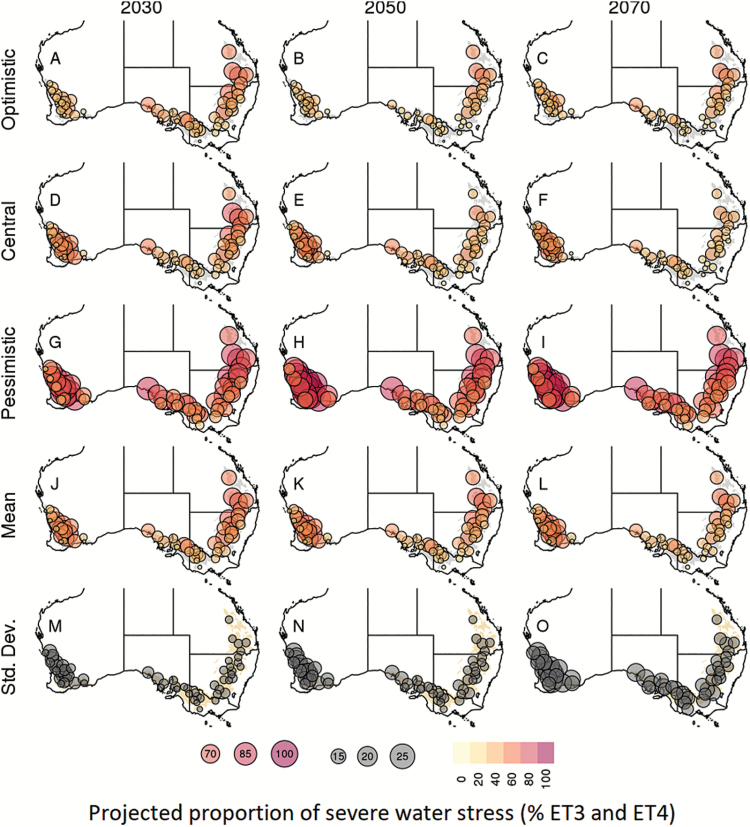
Maps of projected site-level frequency of occurrence of severe water-stress conditions (ET3 and ET4). The optimistic (*CESM1-BGC*), central (*MRI-CGCM3*), and pessimistic (*GFDL-ESM2M*) climate models, as well as the average and SD across the 33 member climate ensemble, are grouped into rows. The 2030, 2050, and 2070 time frames are grouped into columns. The frequencies of occurrence at the different time frames are presented with symbol size and colour (yellow-to-red circles for frequency of occurrence, where circle size and colour indicate magnitude; grey circles for SD, where circle size indicates magnitude). See Supplementary Figs S5–S7 for the projected frequencies of ET1-2-3-4 for 2030, 2050, and 2070.

Despite the wide range in water-stress projection, a consensus arose from the majority of the climate models with a projected increase in severe water-stress conditions in the West (>70% of the models for 2030 onwards), and a projected decrease in the East, South-East, and South of the wheatbelt (>60% of the models for 2030 onwards; Fig. 4F). Across models, the frequency of severe water-stress environments (ET3 and ET4) was projected to increase on average by 5.4, 7.9, and 8.6% in the West by 2030, 2050, and 2070, respectively. In contrast, severe water-stress conditions are projected to change by –5.4, –16.0, and –27.1% across the other regions by 2030, 2050, and 2070, respectively.

While reduced occurrences in water stress were projected by some models (especially in the East, South, and South-East), water stress is projected to remain an important limiting factor for wheat productivity across the whole wheatbelt and for all the time frames tested (Fig. 7; Supplementary Figs S5–S9). Overall, the frequencies of ET1:ET2:ET3:ET4 are projected to change from 28:29:31:12% (1990 levels) to 28:28:31:13% in 2030, 30:27:29:13% in 2050, and 35:25:26:14% in 2070 (Supplementary Figs S5–S7). Note that combined rates of severe water stress (ET3 and ET4) do not drop below 40%.

## Discussion

### Changes in climate are projected to change the spatial distribution of severe water stress

There is general consensus across the climate models of the CMIP5 ensemble that temperatures for the Australian wheat season (May to October) will increase across the wheatbelt in the coming decades (Fig. 4A). Less agreement was found for projected changes in cumulated rainfall. These results are consistent with national analyses showing that the CMIP5 climate models broadly agree on future temperatures, but do not agree on future rainfall patterns in Australia (Reisinger *et al.*, 2014). The 33 climate models studied here provide bounds on the likely distribution of future environmental conditions. In addition, rather than framing this study around the projected climate variations, crop modelling was used to investigate water-stress patterns that wheat crops are projected to experience for those climate variations. These patterns, which account for the impact of variation in temperature, rainfall, and CO_2_ on crops, varied significantly across the climate models analysed, even for projections by 2030.

For both historical and future climate scenarios, water-stress patterns exhibited significant spatial variability across the wheatbelt. This is not surprising given the wide variety of soil and climatic conditions across the Australian wheatbelt (e.g. Potgieter *et al.*, 2002), and is consistent with the water-stress characterizations reported by Chenu *et al.* (2013). However, areas of consensus emerged across the climate models for broad areas of the wheatbelt. In particular, the West of the wheatbelt was projected to experience significant increases in severe water-stress conditions, while the East, South-East, and South of the wheatbelt were projected to experience decreases in severe water-stress conditions, as previously found in an analysis of the north-eastern part of the wheatbelt (Lobell *et al.*, 2015). It is interesting to note that crops in the West and South of the wheatbelt, where in-season rainfall dominates, have a similar frequency of severe water stress in baseline climate (Fig. 4F), but are projected to be affected differently by future climates. Such changes were primarily driven by a divergence in future rainfall projections (Fig. 4B) and may arise from the different levels of regional impact of the Indian Ocean Dipole (IOD), Southern Australian Mode (SAM), subtropical ridge, and possibly the El Niño Southern Oscillation (ENSO) (e.g. Ashok, 2003; Meyers, 2007; Larsen, 2008; Williams and Stone, 2009; Nicholls, 2010). In contrast, while the South and South-East had similar frequencies of rainfall change (Fig. 4B), temperature increases were greater in the South-East (Fig. 4A), leading to a greater south-eastern reduction in the projected occurrence of severe water stress (Fig. 4F).

**Fig. 9.**
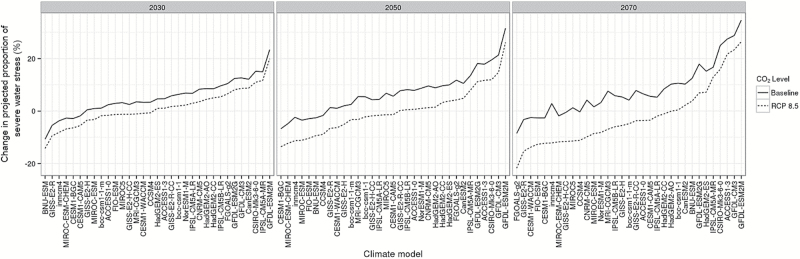
Change in projected occurrence of severe water-stress environment types (ET3 and ET4) across the CMIP5 climate model ensemble for baseline (solid line) and projected (dotted line) CO_2_ levels by 2030, 2050, and 2070. The simulated results correspond to national averages obtained for each climate model, in two configurations: (i) with CO_2_ values fixed at the 1990 concentration; and (ii) with CO_2_ values set according to the projections of RCP8.5. The 1990 baseline CO_2_ level was 354 ppm, while the RCP8.5 levels were 449, 541, and 677 ppm for 2030, 2050, and 2070, respectively. Note the significant reduction in water-stress conditions with the higher CO_2_ concentrations, which is related to increased transpiration efficiency.

The decreases in severe water-stress occurrence projected for the East, South-East, and South were not necessarily due to an increase in rainfall (e.g. Fig. 4B, F), which only occurred for a minority of locations and models (e.g. Figs 4B, 6). Rather, these decreases were related to (i) higher temperatures, which shorten the crop cycles (Sadras and Monzon, 2006; Zheng *et al.*, 2012), and (ii) increased CO_2_ levels which improve crop growth rates through increased transpiration efficiency and radiation use efficiency (van der Kooi *et al.*, 2016). To test the impact of high CO_2_ on projected water-stress patterns, the simulations were re-run with the historical CO_2_ concentration (baseline of 354 ppm). As expected, higher CO_2_ concentrations substantially decreased water-stress occurrence, with more frequent severe water stress identified using current CO_2_ levels than for the higher projected levels of RCP8.5 (Fig. 9). This effect increased with the CO_2_ level, from 2030 to 2070. Without the increase in CO_2_, an overall decrease in severe water stress was projected by relatively few climate models. Hence, by using the RCP8.5 ‘business as usual’ CO_2_ scenario in this study, the projected incidence of severe water-stress environment types was decreased compared with the other, more conservative CO_2_ emission scenarios, particularly in the long-term time frames. The significant impact of CO_2_ also increased the uncertainty of long-term projections, as economic, technological, and political dynamics may result in significantly different CO_2_ concentrations relative to RCP8.5.

Overall, the consensus across climate models was for a projected decrease in yield in the coming decades in the South and West, and a yield increase in the East (Supplementary Figs S8–S9), while previous studies mostly projected decreases in yield for the different Australian regions (e.g. Luo *et al.*, 2005; Ludwig and Asseng, 2006; Yang *et al.*, 2014), using other assumptions in terms of locations, management practices, and, most importantly climate projections or climatic factors. Accordingly to the results found here, yield was also projected to increase in eastern Australia by Wang *et al.* (2009). However, these authors projected yield increases in the South, which was only found for a minority of climate models here. It is important, however, to note that crop modelling studies, including this one, often do not account for heat stress on grain set and grain development, meaning that yield projections need to be interpreted carefully. The results of this study nevertheless highlight that a decrease in severe water-stress occurrence is not necessarily associated with an increase in yield (e.g. in the South; Supplementary Figs S8–S9; Anwar *et al.*, 2007), as factors other than water deficit are important to consider (e.g. increased temperature resulting in shorter crop cycle).

### Limits of future projections

This study aimed to characterize the likely water-stress conditions indicated by the CMIP5 climate ensemble should RCP8.5 become a reality. Future CO_2_ concentrations are highly uncertain, particularly later this century, as they are dependent on a range of geological, economic, and technological factors and decisions that are difficult to anticipate (IPCC, 2014, p. 56). The use of RCP8.5 impacts our results as discussed above (Fig. 9). RCP8.5 is the high end of CO_2_ emissions, but also the most justifiable emissions scenario for short- and mid-term projections time frames, which are critical for breeding and other industry adaptations (Chapman *et al.*, 2012). However, note that this study could be considered as conservative, since other CO_2_ scenarios are likely to project more extreme water-stress patterns, assuming similar temperature and rainfall patterns.

Simulated results highlighted disagreement among the CMIP5 climate models. Variations across climate models were often greater than differences between the 2030, 2050, and 2070 time scales (Fig. 4). This was generally the case for projected rainfall and water-stress frequency (Fig. 4B, C, D, F). While substantial variations exist among climate models for projected temperatures (Fig. 4A), variations are greater for projected rainfall (Fig. 4B), which is harder to capture (IPCC, 2014). Overall, such uncertainties impair agricultural studies such as this one. While focusing on the range and consensus values of the CMIP5 ensemble, this study did not weigh individual climate according to their accuracy for Australia. Previous work has considered the CSIRO Mark 3.5 climate model (which ranked within the four most pessimistic climate models here; Fig. 9) as having skill in projected rainfall and temperature across the Australian wheatbelt (Potgieter *et al.*, 2013). Thus, the overall results presented here may underestimate the likelihood of future severe water stress.

The downscaling method used to generate local weather conditions also presents some major limitations, and different methods have been proposed to generate daily climate projections (e.g. Lutz *et al.*, 2016; Wilcke and Bärring, 2016). For the main simulations from this study, future climate scenarios retained the daily historical temperature and rainfall patterns for each site. Daily variations are expected to change along with climate change, particularly with regard to extreme events such as heat waves and floods (IPCC, 2014), which could have major effects on water-stress patterns (Ludwig *et al.*, 2009). In addition, by keeping patterns of rainfall broadly the same, the projected changes in future planting dates reported in this study are, again, likely to be underestimates.

Furthermore, this specific study did not investigate the projected effects of other stresses, such as frost and heat, which impact wheat crops and their management, for exemple changes in sowing dates (Zheng *et al.*, 2012, 2015; Lobell *et al.*, 2015). This study did not account either for potential changes in land use, in management practices such as change in fertilization, or in cultivars with respect to changes in climate (Luo *et al.*, 2009; Chapman *et al.*, 2012; Shabani and Kotey, 2016; Zheng *et al.*, 2016).

### Where to from here?

While the decrease in severe water-stress conditions projected in this study for much of the wheatbelt could be seen as a positive result, care must be taken not to interpret these results as discouraging adaptation efforts, for several reasons. First, as discussed in the previous section, the findings of this study are likely to be conservative. Secondly, the identified reduction in water-stress conditions is in part driven by a reduction in the crop cycle, which means less time to accumulate resources and fill grains (Zheng *et al.*, 2012, 2016). Thirdly, impacts from other factors influenced by climate change, such as heat stress, are likely to influence crop yields in addition to drought stress (Lobell *et al.*, 2015). Fourthly, while decreasing in many locations, frequencies of severe water-stress conditions were projected to remain substantial across the entire wheatbelt (43.9, 42.6, and 40.2% for 2030, 2050, and 2070, respectively; Figs 4F, 7). Finally, the Western region is projected by this study to experience an increase in severe water stress (Figs 4F, 7), and it is both the largest wheat-producing region from Australia (32% of the national production on average; source: Department of Agriculture, ABARES, Australian Crop Report, February 2014, No. 169), and the largest exporting state of the nation (62% of total wheat exports in 2012; source: Australian Bureau of Statistics).

In the Western region, in addition to increased water-stress occurrence, fewer planting opportunities were projected in the coming decades. While growers from this region are increasingly ‘dry sowing’ their crops (i.e. sowing without waiting for rainfall), the climate models project a slight decrease in rainfall during the sowing window (e.g. an average of –7.9, –8.9, and –13.3% for May by 2030, 2050, and 2070, respectively), and early water deficit when plants emerge may become an increasing problem (Fletcher *et al.*, 2015). Furthermore, low yielding crops (≤500 kg ha^−1^) were projected to increase substantially in Western Australia (data not shown). All these factors are likely to affect future crop production in the West for wheat, and probably for other crops as well. Since Western Australia not only produces much of the continent’s wheat, but is also the main producer of winter crops, it is a priority to study the projected impacts of climate change on other crops further, especially in this state.

Adaptation planning can thus be seen as critical for Australia, especially in the West (Asseng and Pannell, 2013; Fischer *et al.*, 2014), where severe water-stress conditions are projected to increase by 5.4, 7.9, and 8.6% by 2030, 2050, and 2070, respectively, reaching a frequency of occurrence of 42.7, 45.2, and 45.9%, respectively. A recent sensitivity analysis for the APSIM wheat model has identified traits related to phenology, root systems, plant architecture, transpiration efficiency, photosynthesis, biomass allocation, and grain filling rate as key potential candidates for crop improvement in Australia (Casadebaig *et al.*, 2016). Ongoing efforts are deployed in physiology, genetics, breeding, and management to improve adaptation to drought (e.g. Richards *et al.*, 2010; Chapman *et al.*, 2012; Kirkegaard *et al.*, 2014), with innovative solutions that utilize multiple methods: phenotyping, genomics, genetics, modelling, and system planning (e.g. Chenu *et al.*, 2009a;Furbank and Tester, 2011; Rebetzke *et al.*, 2013; Zheng *et al.*, 2013; Chapman *et al.*, 2014; Christopher *et al.*, 2014; Richard *et al.*, 2015; Technow *et al.*, 2015).

## Conclusions

Understanding the potential effect of climate change on the Australian wheatbelt is essential for the continued success of the local wheat industry. In order to characterize the occurrence of water-stress conditions across the Australian wheatbelt in the coming decades, extreme projections, as well as consensus, from 33 state-of-the-art climate models were analysed to provide farmers, breeders, and policy makers with valuable information to consider in adaptation planning. Overall, the East, South-East, and South regions of the wheatbelt were projected to experience fewer severe water-stress conditions in the coming decades compared with 1990 baseline levels. In contrast, the Western region of the wheatbelt, which is the largest contributor to Australian wheat exports, was projected to experience an increased frequency in severe water-stress conditions. Where found, reductions in severe water stress were largely driven by shorter crop cycles arising from higher temperatures, and increased transpiration efficiency due to higher CO_2_ concentrations, which overcame the overall reduction in rainfall projected for most locations by most climate models.

The Australian wheat industry has historically adapted to severe water stress with high interannual variability. This study indicates that the local wheat industry will continue to experience the effect of severe water-stress conditions in the coming decades. Australia is a major exporter of wheat, and demand for this crop is projected to keep increasing. Coupled with the 5–15 year lead times involved in breeding new cultivars, this study provides justification for immediate adaptation planning, particularly in the Western region.

## Supplementary Data

Supplementary data are available at *JXB* online.

Table S1. Regions, locations, and soils chosen to represent the Australian wheatbelt.

Fig. S1. Maps of site-level seasonal mean temperatures projected by the optimistic, central, and pessimistic climate models, as well as the average projection and SD across the 33 member climate models ensemble for 2030, 2050, and 2070.

Fig. S2. Maps of site-level seasonal cumulated rainfall projected by the optimistic, central, and pessimistic climate models, as well as the average projection and SD across the 33 member climate model ensemble, for 2030, 2050, and 2070.

Fig. S3. Maps of site-level frequency of years with no planting opportunity projected by the optimistic, central, and pessimistic climate models, as well as the average projection and SD across the 33 member climate model ensemble for 2030, 2050, and 2070.

Fig. S4. Maps of site-level mean planting date projected by the optimistic, central, and pessimistic climate models, as well as the average projection and SD across the 33 member climate model ensemble for 2030, 2050, and 2070.

Fig. S5. Maps of site-level changes in the occurrence of water-stress environment types (ET1, ET2, ET3, and ET4) projected by the optimistic, central, and pessimistic climate models, as well as the average projection and SD across the 33 member climate model ensemble for 2030.

Fig. S6. Maps of site-level changes in the occurrence of water-stress environment types (ET1, ET2, ET3, and ET4) projected by the optimistic, central, and pessimistic climate models, as well as the average projection and SD across the 33 member climate model ensemble for 2050.

Fig. S7. Maps of site-level changes in the occurrence of water-stress environment types (ET1, ET2, ET3, and ET4) projected by the optimistic, central, and pessimistic climate models, as well as the average projection and SD across the 33 member climate model ensemble for 2070.

Fig. S8. Maps of site-level changes in mean crop yield projected by the optimistic, central, and pessimistic climate models, as well as the average and SD across the 33 member climate model ensemble for 2030, 2050, and 2070.

Fig. S9. Projected changes in mean yield for the major wheatbelt areas, across all 33 members of the climate model ensemble for 2030, 2050 and 2070.

## Supplementary Material

Supplementary Material
