# Supplementary material for: Projected impact of future climate on water-stress patterns across the Australian wheatbelt
Source: J Exp Bot. 2017 Nov 25;68(21-22):5907–21. doi: 10.1093/jxb/erx368 (PMC5854138; doi:10.1093/jxb/erx368)
Supplement: Supplementary Material [file erx368_suppl_supplementary_material.pdf]

SUPPLEMENTARY MATERIAL

Region	Location	State	Lat. (°)	Long. (°)	Soil classification	Soil PAWC (mm)
West	Carnamah	WA	-29.69	115.89	Sand	98
	Geraldton	WA	-28.80	114.70	Sand	98
	Badgingarra	WA	-30.34	115.54	Gravel	54
	Corrigin	WA	-32.33	117.87	Deep sandy duplex	74
	Nyabing	WA	-33.54	118.15	Clay	135
	Cunderdin	WA	-31.66	117.25	Deep sandy duplex	74
	WonganHills	WA	-30.84	116.73	Deep loamy duplex	90
	Katanning	WA	-33.69	117.56	Deep sandy duplex	74
	Wandering	WA	-32.68	116.68	Gravel	54
	Northam	WA	-31.64	116.67	Loamy earth	135
	Bencubbin	WA	-30.81	117.86	Sandy earth	112
	Dalwallinu	WA	-30.28	116.66	Sand	98
	Kellerberrin	WA	-31.62	117.72	Sandy earth	112
	Merredin	WA	-31.48	118.28	Shallow loamy duplex	101
	Morawa	WA	-29.21	116.01	Deep loamy duplex	90
	Mullewa	WA	-28.54	115.51	Sandy loam	34
	SouthernCross	WA	-31.23	119.33	Gravel	54
	Narembeen	WA	-32.07	118.40	Loamy earth	135
	Esperance	WA	-33.61	121.78	Deep sandy duplex	74
	Hyden	WA	-32.44	118.90	Deep sandy duplex	74
	LakeGrace	WA	-33.10	118.46	Shallow sandy duplex	57
	SalmonGums	WA	-32.99	121.62	Shallow sandy duplex	57
	Ravensthorpe	WA	-33.58	120.05	Deep sandy duplex	74
South	Minnipa	SA	-32.84	135.15	Red light sandy clay loam	90
	Ceduna	SA	-31.90	133.42	Grey calcareous sandy loam	64
	Rudall	SA	-33.69	136.27	Grey calcareous loamy sand	87
	Cummins	SA	-34.27	135.73	Dark loamy clay	105
	Balaklava	SA	-34.14	138.42	Sandy loam	72
	Roseworthy	SA	-34.53	138.69	Dark brown cracking clay	94
	PortPirie	SA	-33.17	138.01	Loamy sand	139
	Loxton	SA	-34.44	140.60	Loamy sand	134
	Waikerie	SA	-34.18	139.98	Hypercalcic calcarosol	82
	Pinnaroo	SA	-35.26	140.91	Loamy sand	79
	Walpeup	SA	-35.12	142.00	Loamy sand	134
South-East	Urana	NSW	-35.33	146.03	Clay loam	172
	Yanco	NSW	-34.61	146.42	Brown sodosol	191
	WaggaWagga	NSW	-35.16	147.46	Red sodosol	181
	Longerenong	VIC	-36.67	142.30	Clay	179
	Dookie	VIC	-36.37	145.70	Loam	168
	LakeBolac	VIC	-33.10	118.46	Sandy clay loam	151
	Hopetoun	VIC	-35.73	142.37	Loamy sand	153

	Glenlee	VIC	-36.26	141.86	Clay	148
	Birchip	VIC	-35.98	142.92	Clay loam	99
East	Merriwagga	NSW	-33.92	145.52	Sandy loam	165
	Parkes	NSW	-33.14	148.16	Sandy clay loam	196
	Gilgandra	NSW	-31.71	148.66	Brown dermosol	128
	Condobolin	NSW	-33.07	147.23	Sandy loam	132
	Dubbo	NSW	-32.24	148.61	Red dermosol	142
	Nyngan	NSW	-31.55	147.20	Sandy clay loam	219
	Gunnedah	NSW	-30.98	150.25	Black vertosol	272
	Wellington	NSW	-32.80	148.80	Sandy clay loam	101
	Narrabri	NSW	-30.34	149.76	Grey vertosol	233
	Moree	NSW	-29.48	149.84	Grey vertosol	194
	Walgett	NSW	-30.04	148.12	Grey vertosol	194
	Coonamble	NSW	-30.98	148.38	Sandy clay	181
	Roma	QLD	-26.57	148.79	Brown vertosol	119
	Meandarra	QLD	-27.33	149.88	Grey vertosol	201
	Goondiwindi	QLD	-28.55	150.31	red brown	159
	Dalby	QLD	-27.18	151.26	Grey vertosol	206
	Emerald	QLD	-23.53	148.16	Black vertosol	134

Tab. S1 Regions, locations and soils chosen to represent the Australian wheatbelt (based on Chenu *et al.*, 2013). Longitude (“Lon.”) and latitude (“Lat.”) are given for each location. The potential available soil water capacity (PAWC) corresponds to the potential amount of water that a wheat crop can extract (i.e., up to the potential crop rooting depth).

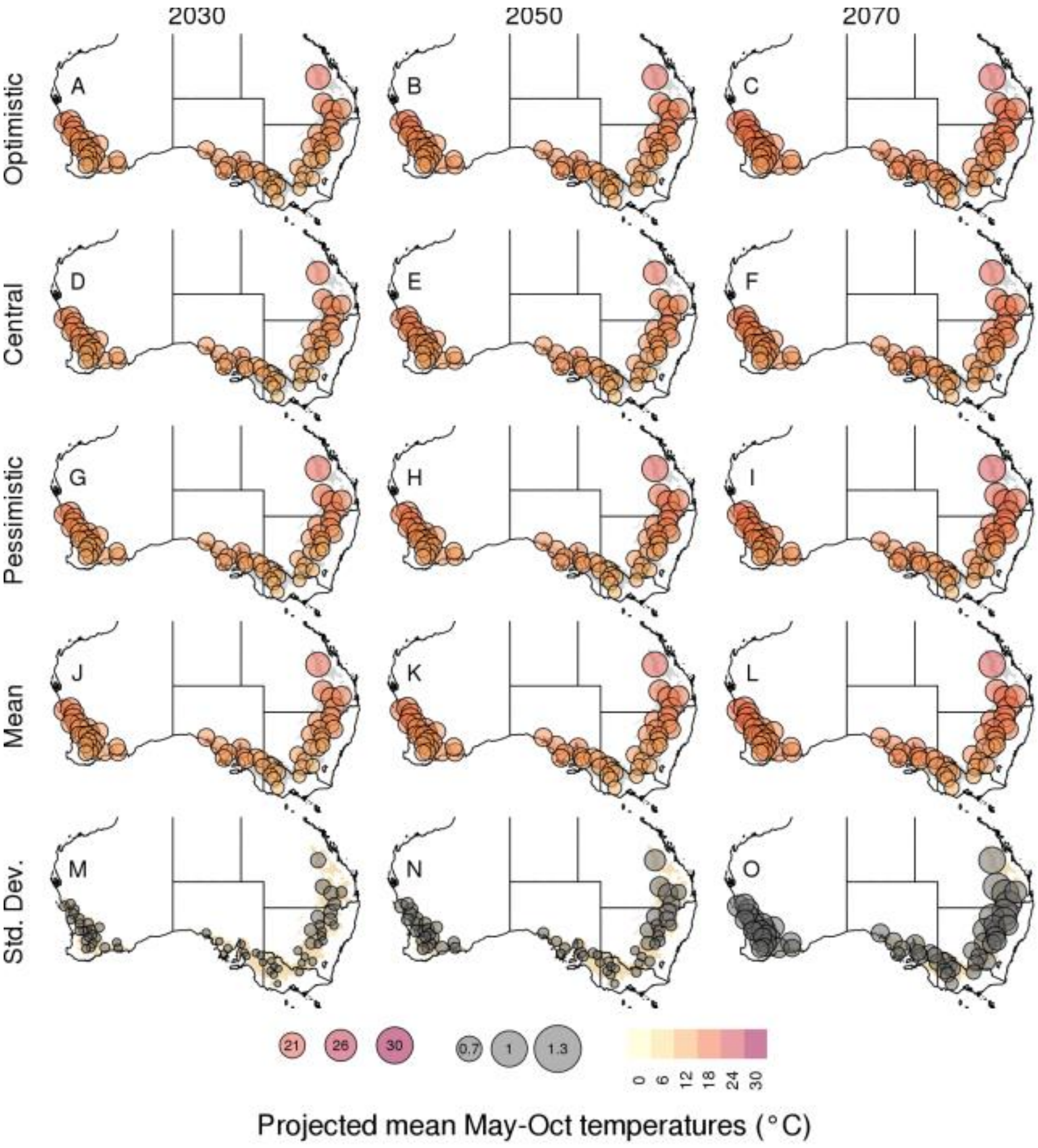

Fig. S1 Maps of site-level seasonal mean temperatures projected by the optimistic (*CESM1-BGC*), central (*MRI-CGCM3*), and pessimistic (*GFDL-ESM2M*) climate models, as well as the average projection and standard deviation across the 33 member climate models ensemble (rows) for 2030, 2050 and 2070 (columns). Mean temperatures were calculated for 1-May to 31-Oct, and are presented with symbol size and colour (yellow-to-red circles for increasing temperature; grey circles for standard deviation). See Fig. 5 for projected changes since 1990.

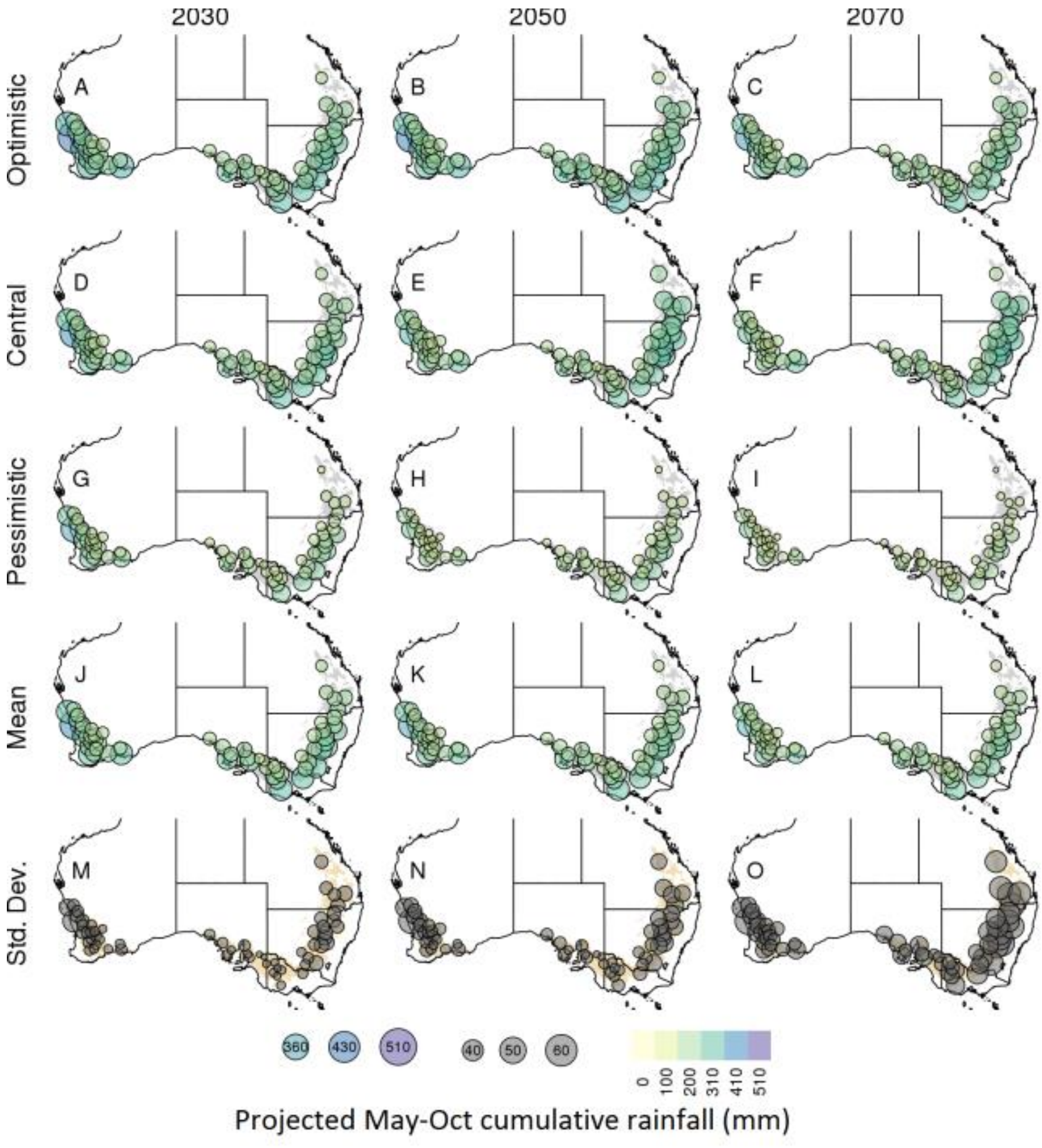

Fig. S2 Maps of site-level seasonal cumulated rainfall projected by the optimistic (*CESM1-BGC*), central (*MRI-CGCM3*), and pessimistic (*GFDL-ESM2M*) climate models, as well as the average projection and standard deviation across the 33 member climate model ensemble (rows), for 2030, 2050 and 2070 (columns). Average cumulated rainfall was calculated for 1-May to 31-Oct, and is presented with symbol size and colour (yellow-to-purple circles for increasing rainfall; grey circles for standard deviation). See Fig. 6 for projected changes since 1990.

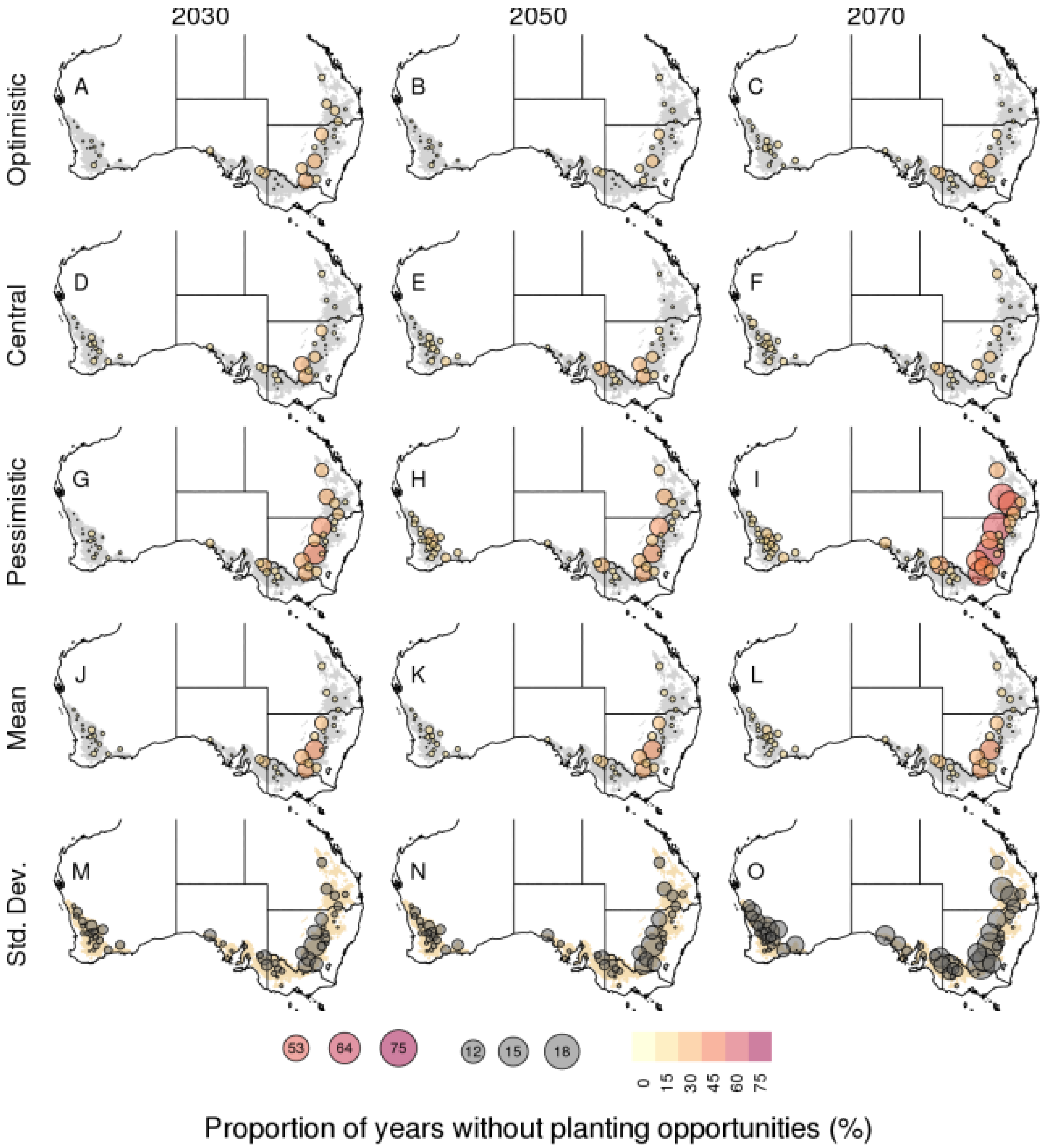

Fig. S3 Maps of site-level frequency of years with no planting opportunity projected by the optimistic (*CESM1-BGC*), central (*MRI-CGCM3*), and pessimistic (*GFDL-ESM2M*) climate models, as well as the average projection and standard deviation across the 33 member climate model ensemble (rows) for 2030, 2050 and 2070 (columns). Data are presented with symbol size and colour (yellow-to-red circles for increasing frequencies; grey circles for standard deviation).

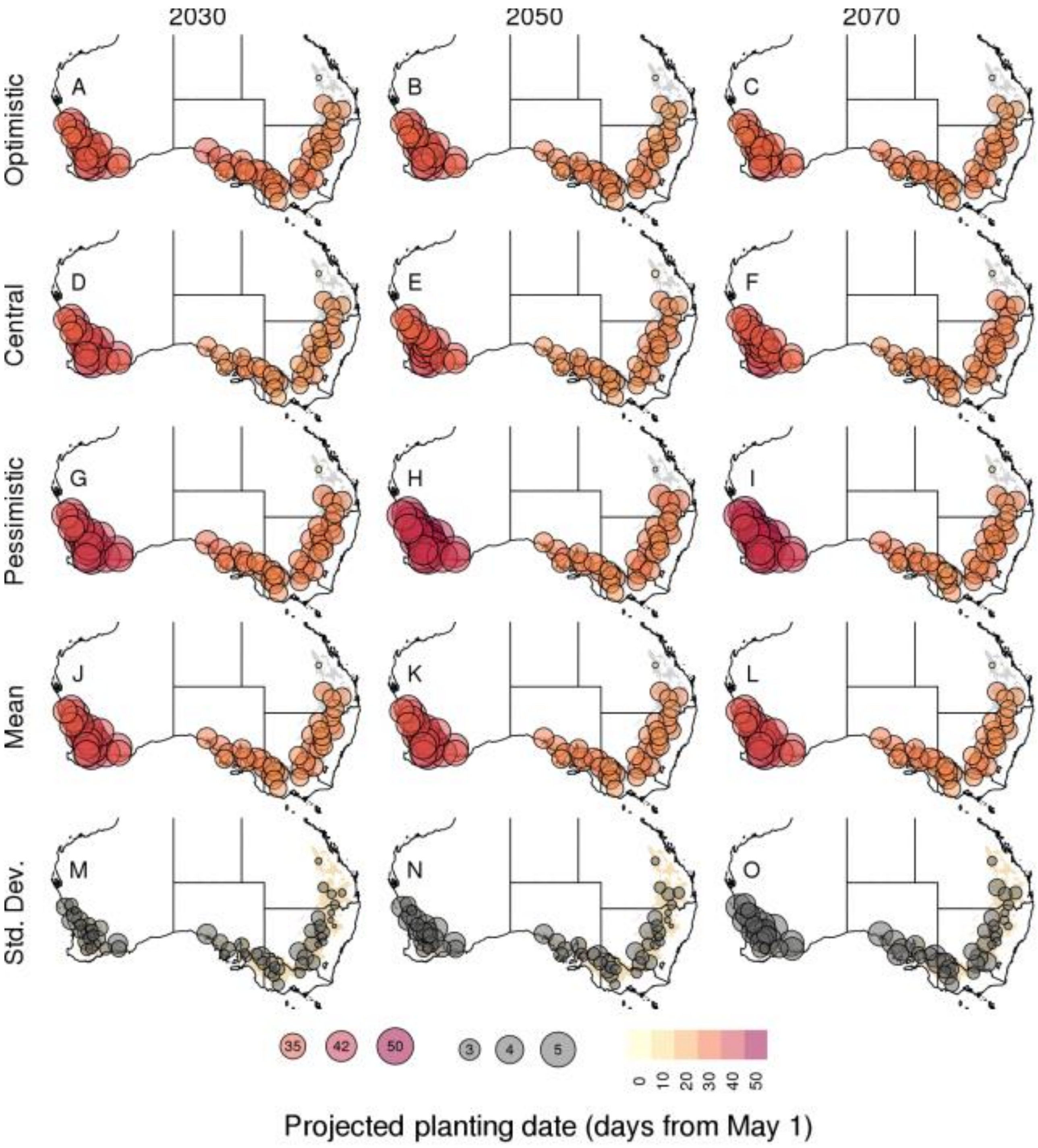

Fig. S4 Maps of site-level mean planting date projected by the optimistic (*CESM1-BGC*), central (*MRI-CGCM3*), and pessimistic (*GFDL-ESM2M*) climate models, as well as the average projection and standard deviation across the 33 member climate model ensemble (rows) for 2030, 2050 and 2070 (columns). Data are presented with symbol size and colour (yellow-to-red circles for increasing delays in planting date; grey circles for standard deviation).

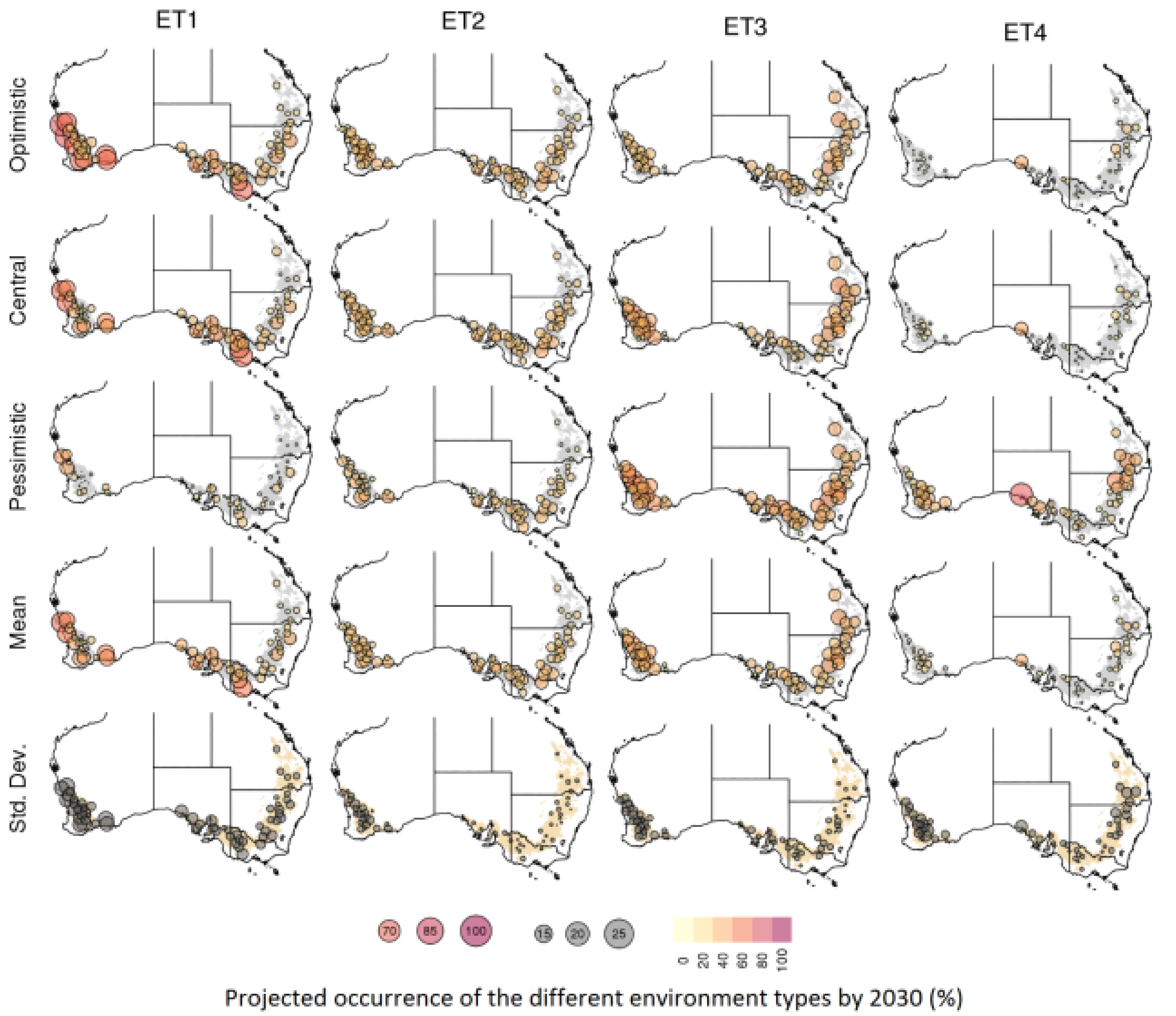

Fig. S5 Maps of site-level changes in the occurrence of water-stress environment types (ET1, ET2, ET3 and ET4) projected by the optimistic (*CESM1-BGC*), central (*MRI-CGCM3*), and pessimistic (*GFDL-ESM2M*) climate models, as well as the average projection and standard deviation across the 33 member climate model ensemble (rows) for 2030. Frequencies are presented with symbol size and colour (yellow-to-red circle for increasing frequencies of occurrence; grey circle for standard deviation).

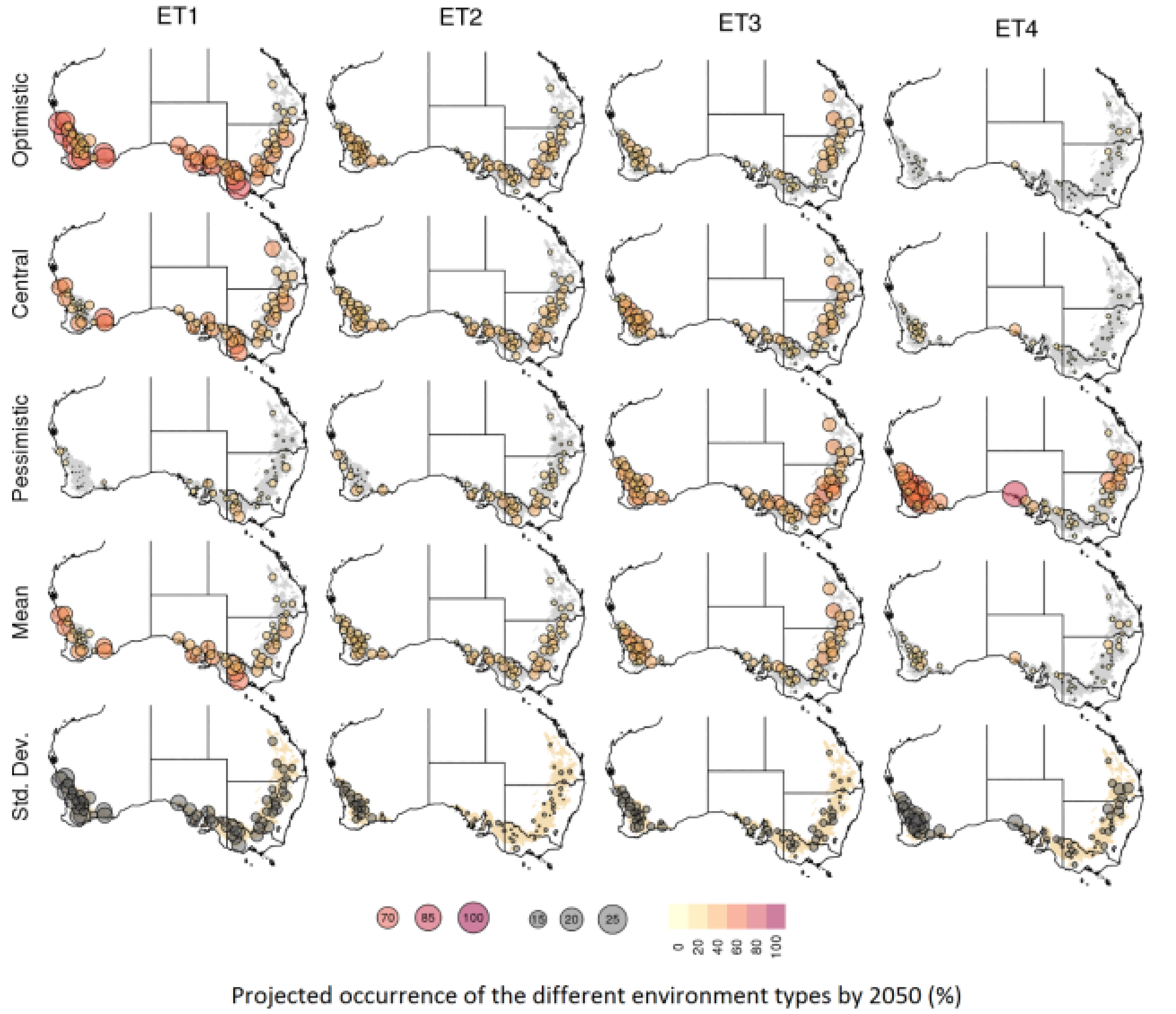

Fig. S6 Maps of site-level changes in the occurrence of water-stress environment types (ET1, ET2, ET3 and ET4) projected by the optimistic (*CESM1-BGC*), central (*MRI-CGCM3*), and pessimistic (*GFDL-ESM2M*) climate models, as well as the average projection and standard deviation across the 33 member climate model ensemble (rows) for 2050. Frequencies are presented with symbol size and colour (yellow-to-red circle for increasing frequencies of occurrence; grey circle for standard deviation).

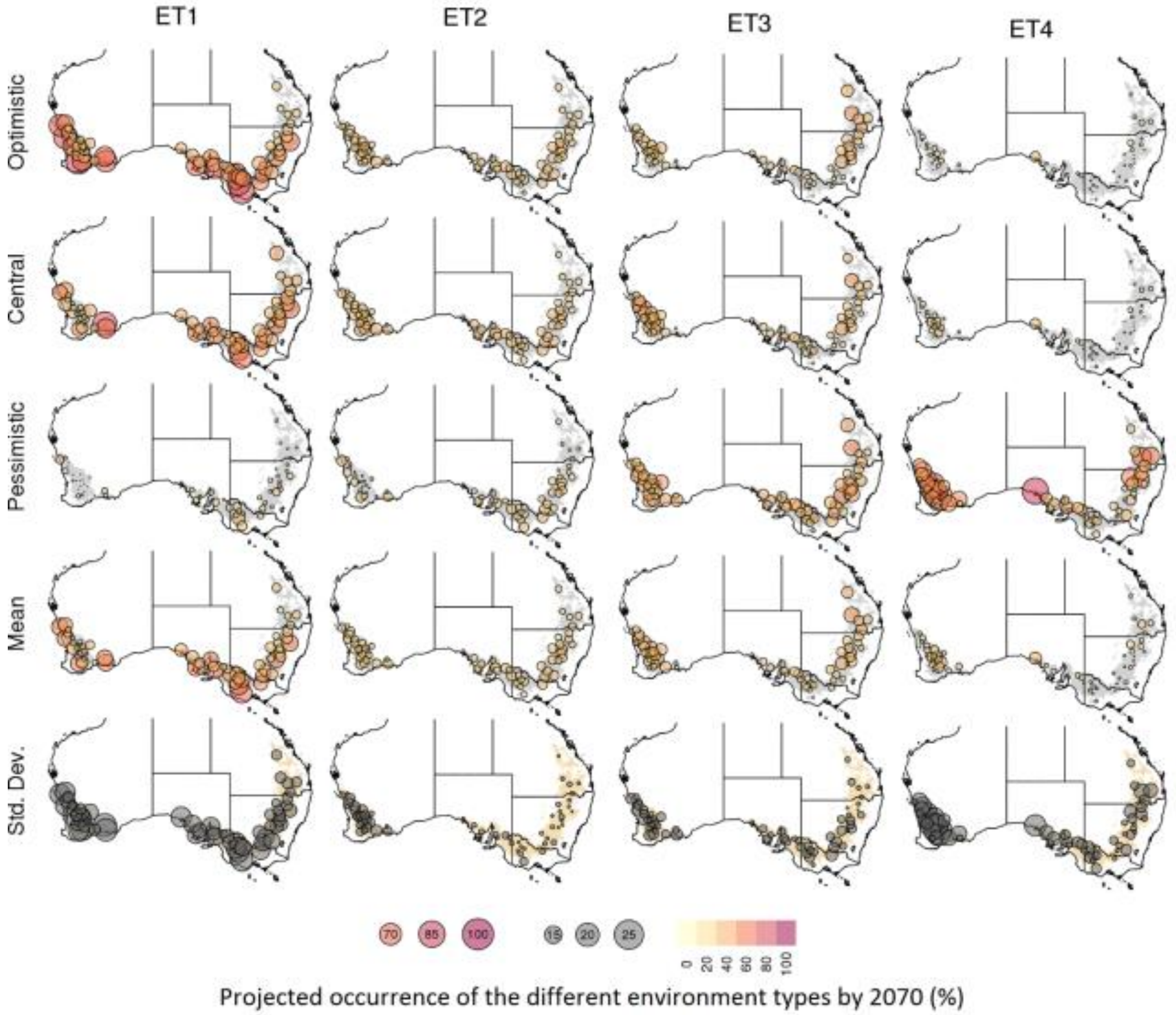

Fig. S7 Maps of site-level changes in the occurrence of water-stress environment types (ET1, ET2, ET3 and ET4) projected by the optimistic (*CESM1-BGC*), central (*MRI-CGCM3*), and pessimistic (*GFDL-ESM2M*)climate models, as well as the average projection and standard deviation across the 33 member climate model ensemble (rows) for 2070. Frequencies are presented with symbol size and colour (yellow-to-red circle for increasing frequencies of occurrence; grey circle for standard deviation).

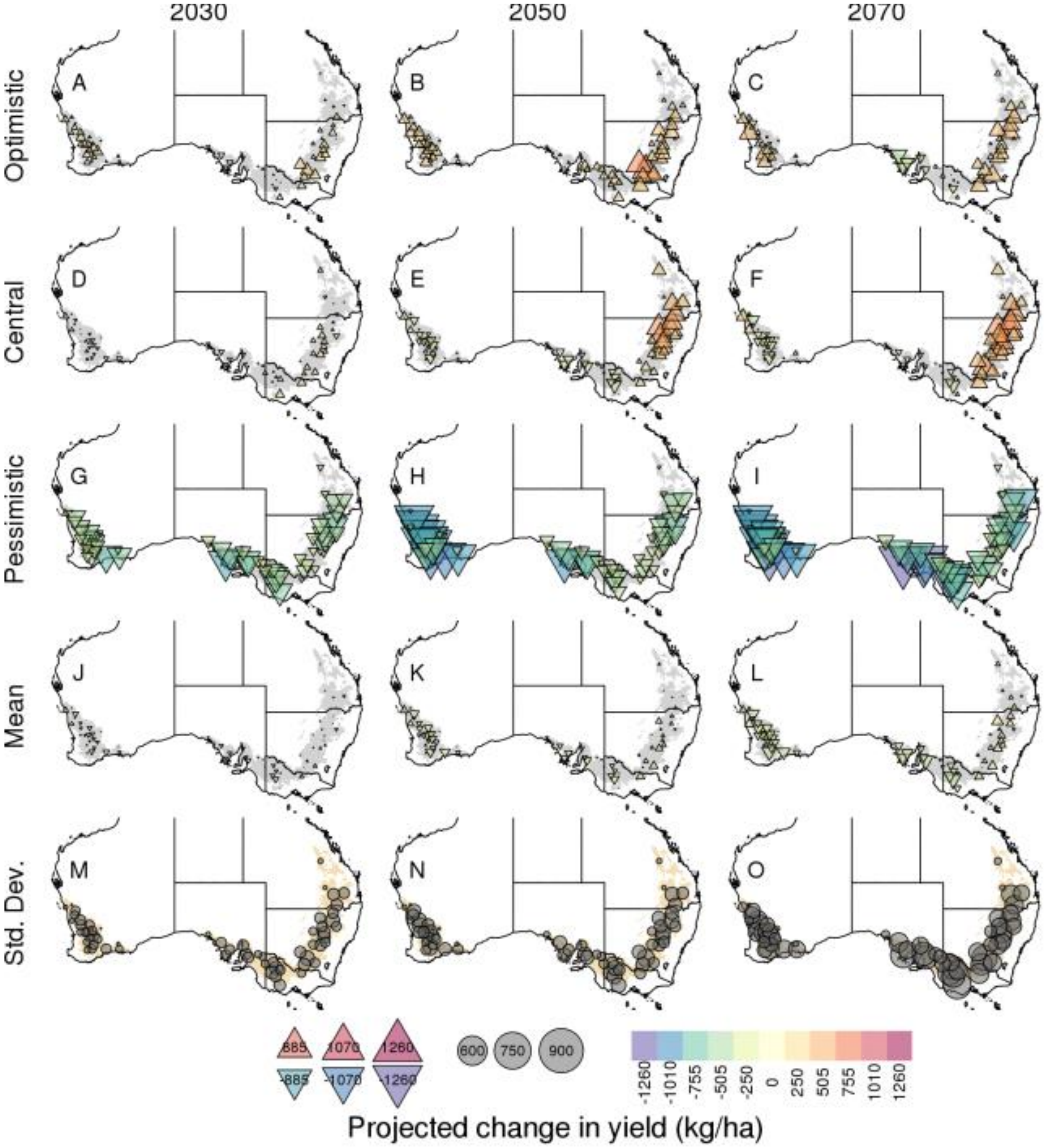

Fig. S8 Maps of site-level changes in mean crop yield projected by the optimistic (*CESM1-BGC*), central (*MRI-CGCM3*), and pessimistic (*GFDL-ESM2M*) climate models (top three rows). The climate model ensemble averages and standard deviations are also shown (bottom two rows). Values are shown for the 2030, 2050 and 2070 timeframes (columns). Changes were calculated with respect to the 1990 (baseline) values, and are represented by symbols and colour (up-triangles for increased values, down-triangles for decreased values, with colour and symbol size indicating magnitude; grey circles for standard deviations). Note that these simulations do not include the effect of heat stress on grain setting and grain development.

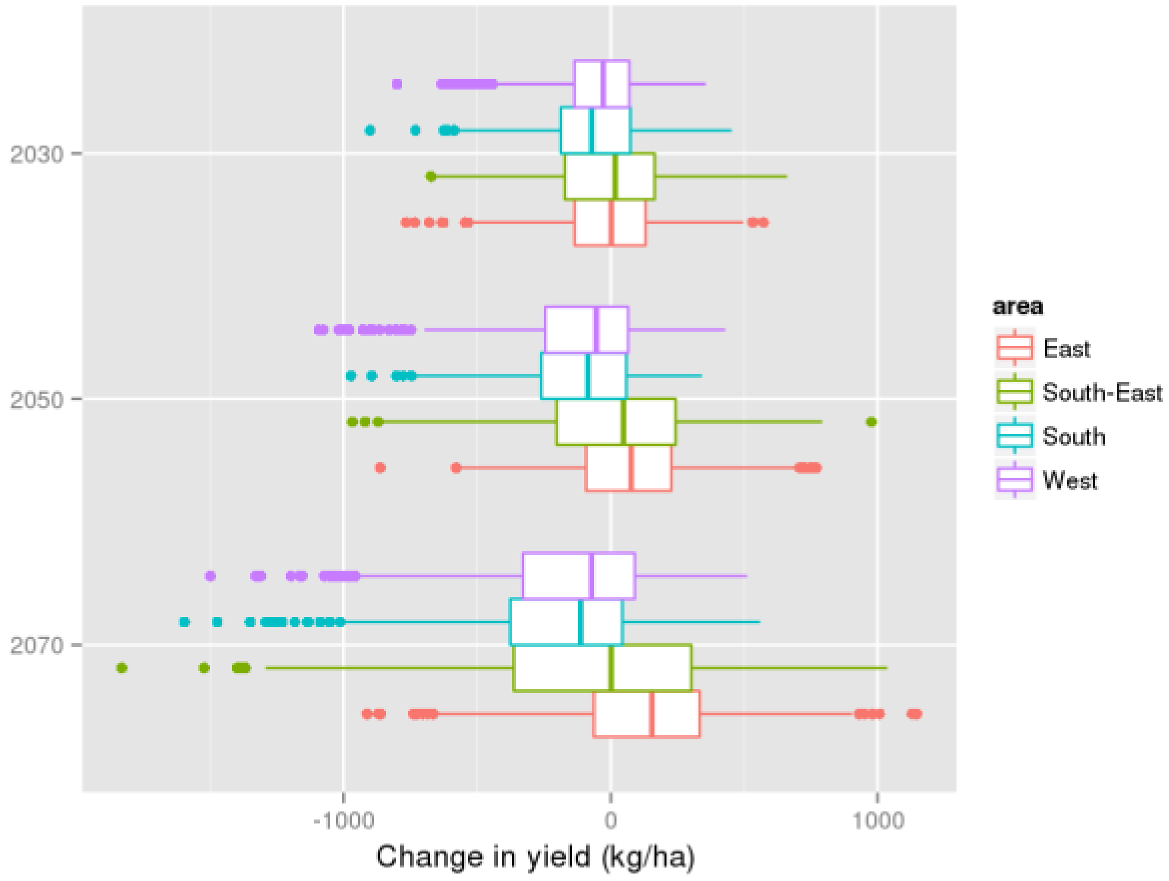

Fig. S9 Projected changes in mean yield for the major wheatbelt areas, across all 33 members of the climate model ensemble, for 2030, 2050 and 2070. The lines within the boxes represent the median value of the data, the higher value edge of the boxes represent the 75th percentile, and the lower value edges represent the 25th percentile. The whiskers correspond to 1.5 times the interquartile range, or to the most extreme observed value (whichever is smallest). The dots represent values outside of 1.5 times the interquartile range. Note that simulations do not include the effect of heat stress on grain setting and grain development.
